# Applications and Advances in Electronic-Nose Technologies

**DOI:** 10.3390/s90705099

**Published:** 2009-06-29

**Authors:** Alphus D. Wilson, Manuela Baietto

**Affiliations:** 1 Southern Hardwoods Laboratory, Center for Bottomland Hardwoods Research, Southern Research Station, USDA Forest Service, P.O. Box 227, Stoneville, Mississippi, 38776, USA; 2 Department of Crop Science, University of Milan,Via Celoria 2, 20133, Milan, Italy; E-Mail: manuela.baietto@unimi.it

**Keywords:** artificial olfaction, conducting polymers, electronic aroma detection, e-nose

## Abstract

Electronic-nose devices have received considerable attention in the field of sensor technology during the past twenty years, largely due to the discovery of numerous applications derived from research in diverse fields of applied sciences. Recent applications of electronic nose technologies have come through advances in sensor design, material improvements, software innovations and progress in microcircuitry design and systems integration. The invention of many new e-nose sensor types and arrays, based on different detection principles and mechanisms, is closely correlated with the expansion of new applications. Electronic noses have provided a plethora of benefits to a variety of commercial industries, including the agricultural, biomedical, cosmetics, environmental, food, manufacturing, military, pharmaceutical, regulatory, and various scientific research fields. Advances have improved product attributes, uniformity, and consistency as a result of increases in quality control capabilities afforded by electronic-nose monitoring of all phases of industrial manufacturing processes. This paper is a review of the major electronic-nose technologies, developed since this specialized field was born and became prominent in the mid 1980s, and a summarization of some of the more important and useful applications that have been of greatest benefit to man.

## 1. Introduction

The sensor technology of artificial olfaction had its beginnings with the invention of the first gas multisensor array in 1982 [1]. Advances in aroma-sensor technology, electronics, biochemistry and artificial intelligence made it possible to develop devices capable of measuring and characterizing volatile aromas released from a multitude of sources for numerous applications. These devices, known as electronic noses, were engineered to mimic the mammalian olfactory system within an instrument designed to obtain repeatable measurements, allowing identifications and classifications of aroma mixtures while eliminating operator fatigue [2–7]. Unlike other analytical instruments, these devices allow the identification of mixtures of organic samples as a whole (identifiable to a source that released the mixture) without having to identify individual chemical species within the sample mixture [2,8,9]. Hundreds of different prototypes of artificial-nose devices have been developed to discriminate complex vapor mixtures containing many different types of volatile organic compounds (VOCs) [10,11]. These prototypes collectively represent various electronic aroma detection (EAD) technologies that utilize different sensor types including metal-oxide [12–14], semiconductive polymers [15–16], conductive electroactive polymers [9,17–19], optical [20], surface acoustic wave [20] and electrochemical gas sensors [21].

An electronic nose system typically consists of a multisensor array, an information-processing unit such as an artificial neural network (ANN), software with digital pattern-recognition algorithms, and reference-library databases [8,17,22–24]. The cross-reactive sensor array is composed of incrementally-different sensors chosen to respond to a wide range of chemical classes and discriminate diverse mixtures of possible analytes. The output from individual sensors are collectively assembled and integrated to produce a distinct digital response pattern. Identification and classification of an analyte mixture is accomplished through recognition of this unique aroma signature (electronic fingerprint) of collective sensor responses. The identity of a simple or complex mixture represented by a unique aroma signature pattern may be determined without having to separate the mixture into its individual components prior to or during analysis. A reference library of digital aroma signature patterns for known samples is constructed prior to analysis of unknowns. The ANN is configured through a learning process (neural net training) using pattern recognition algorithms that look for differences between the patterns of all analyte types included in the reference library. This process continues until a previously selected level of discrimination is met. The results are validated and assembled into the reference library to which unknown samples can be compared. Identification of unknowns is based on the distribution of aroma attributes or elements that the analyte pattern has in common with patterns present in databases of the reference library.

Most applications of EAD technologies hitherto have been in industrial production, processing, and manufacturing [10,15,19,25–28]. Some of the more common manufacturing applications have been in quality control and grading, product uniformity and consistency, processing controls, gas leak detection and environmental effluents monitoring [10,11,29–34]. Applications are continuously being developed in many new areas of applied research such as for volatile emissions assessments, homeland security, environmental protection, biomedical diagnoses, personnel safety, and in product-development research. This paper summarizes some theoretical aspects of electronic-nose technologies by describing and comparing some of the basic types of e-nose technologies that have been developed and by reviewing the wide variety and categories of e-nose applications that have been discovered within the past twenty years, particularly those that have been most useful and benefited man in diverse ways.

## 2. The Role of Aroma in Human Society and Commerce

The sense of smell has long played a fundamental role in human development and biosocial interactions. Consequently, the olfactory sense has become a key element in the development of many commercial industries that manipulate the aroma properties of their manufactured goods in order to improve product appeal, quality, and consistency so that consumers quickly identify with individual brands having unique scents. A wide diversity of examples ranging from the bouquet of wines and cuisine, perfumes and colognes added to personal health-care products, and scents applied to product packaging are obvious paradigms demonstrating the importance of aroma qualities in industrial manufacturing and commercial trade. Similarly, spices have been used throughout human history to enhance the flavor of foods and scent the air with aromatic pot-pourris; other examples of products used and valued for their aromatic characteristics. Indeed, spices were once among the most valued commodities for trade in ancient times and considered sufficiently valuable alone to justify the opening of new commercial trade routes throughout the world. Thus, aroma characteristics have contributed immensely to the value and appeal of many commercial products, and have largely determined what consumers are willing to pay for many manufactured goods. As a result, research and quality control of aroma characteristics during product manufacturing has become of paramount importance in industrial production operations because product consistency is essential for maintaining consumer brand recognition and satisfaction. This importance of product aroma characteristics has been repeatedly demonstrated by devastating losses in corporate sales and market share that typically occur when manufacturing changes are made to product aroma and flavor characteristics.

Despite the importance of the olfactory sense to mankind, the sense of smell in man is often considered the least refined of the human senses, far less sensitive than that of other animals. For example, the human nose possesses only about one million aroma receptors that work in tandem to process olfactory stimuli whereas dogs have about 100 million receptors that distinguish scents at least 100 times more effectively than the average human [10]. Furthermore, the ability to detect chemicals in the environment is critical to the survival of most prokaryotic and eukaryotic organisms. A clear indication of the importance of olfactory systems in higher eukaryotes is the significant proportion (up to 4%) of the genome that is devoted to encoding products used in building olfactory sensory tissues [35]. The relatively low sensitivity and discrimination capabilities of the human nose, coupled with the common occurrence of olfactory fatigue, has led to the need for electronic instruments with sensors capable of performing repeated discriminations with high precision to eliminate human fatigue.

The olfactory sense has long been intimately linked with human emotions and aesthetics, yet previously we have lacked a suitable vocabulary to describe aromas with precision and to quantify aromas in more discrete, consistent terms. As a consequence, past researchers have resorted to the use of relative or comparative terms to describe aromatic materials. The need to more precisely quantify and express the aroma characteristics of VOCs, released as mixtures from specific source types, has made necessary the development of methods and instruments capable of recording unique quantitative and qualitative measurements of headspace volatiles derived from known sources. For these reasons, there has been great interest in the development of electrochemical receptors for detecting aromas of complex vapor mixtures.

### 2.1. Aroma Types and Characteristics

Aromas are simple to complex mixtures of volatile compounds present in the air at concentration that may be detected by animals through the sense of olfaction. Aromas sometimes have been referred to as “smells” or “odors” when a particular connotation referring to the pleasantness or unpleasantness of an aroma is being expressed. In some cases, the aroma is composed of a single chemical compound, while in others only a few compounds may be present of which only one may be the dominant or principal component. However, an aroma derived from organic sources in most cases may be composed of hundreds of different compounds all of which contribute to the unique qualities and characteristics of the typical aroma. The effect of even subtle changes in the relative amounts of chemical species within an aroma mixture often can be detected by the human nose as a change in odor by trained panel experts, but changes in odorless materials are not detectable. Nevertheless, the electronic nose often has the advantage of detecting certain odorless compounds that are not detectable by the human nose.

Aromas in general are characterized by four quantifiable qualitative dimensions: threshold, intensity, quality, and hedonic assessment. The detection threshold value is defined as the lowest concentration of aromatic compounds at which human subjects can detect the existence of the aroma [36]. The detection threshold is determined by diluting the aroma to the point where 50% of the test population or human panel can no longer detect the aroma [37]. Intensity refers to the perceived strength of the aroma sensation, and increases as a function of concentration. Quality is the third dimension and is usually expressed by the use of descriptor types, or common-use words that describe the aroma by associating it to the aroma qualities of known substances; and usually referring to aromas released from plants or plant parts. McGinley and McGinley [38] proposed eight aroma groups with examples of descriptor types, representative of each group, as follows: (1) earthy aromas (musty, moldy, musk, stale, grassy, herbal, woody), (2) floral aromas (fragrant, flowery, perfume, eucalyptus, lavender), (3) fruity aromas (citrus, orange, lemon, apple, pear, pineapple, strawberry), (4) spicy aromas (cinnamon, mint, peppermint, onion, dill, garlic, pepper, cloves, vanilla, almond, pine), (5) fishy aromas (fishy, prawns, amine), (6) sewage aromas (septic, putrid, rancid, sulfurous, rotten, decayed, cadaverous, foul, sour, pungent, burnt, swampy), (7) medicinal aromas (disinfectant, phenol, camphor, soapy, ammonia, alcohol, ether, anesthetic, menthol), and (8) chemical aromas (solvent, aromatic, varnish, turpentine, petroleum, creosote, tar, oily, plastic). The hedonic assessment dimension is associated with the relative pleasantness or unpleasantness of the aroma. Hedonic assessment may be quantified using scaled values that range from 1 (completely dislike) to 10 (very good, pleasant, and agreeable) or through objective judgments (excellent, terrible) using descriptor terms indicating relative satisfaction or agreeableness of the aroma (from very pleasant to completely unpleasant).

Aromatic compounds usually have relatively low molecular masses ranging between 30 and 300 Da (g mol^−1^). At room temperature, molecules heavier than this generally have vapor pressures too low to be aromatic. The volatility of molecules is determined by the strength of bonds between them with non-polar molecules being more volatile than polar ones. In fact, most aromatic molecules have no more than one or two polar functional groups because molecules with more polar functional groups generally are not volatile. Volatile compounds frequently contain an oxygen moiety, although nitrogen and sulfur moieties also may be present [39].

Aromatic compounds are mainly characterized by their chemical structural and constituent functional groups, such as heterocyclic systems, double bonds and aromatic rings that contribute to the overall shape of the molecule and produce a particular aroma or flavor sensation. To the common senses, these functional groups may be found in compounds contained within particular foods or drinks [21]. Aromatic compounds also often have delocalized conjugated л-electron structure typical of the benzene ring. However, many other compounds of unknown chemical structure are generically referred to as being aromatic because of their volatile nature or particular aromas. Many types of aromatic compounds are of particular importance in research areas dealing with fragrance and flavor chemistry because many are naturally-occurring aromas and flavors.

The food, beverage and perfume industries that manage and manipulate product aromas have consistently tried to name and classify aromas. Linneaus first proposed seven primary aromas: aromatic, fragrant, musky, garlicky, goaty, repulsive and nauseating [40,41]. This observation was in accordance with psychological studies that suggested the human mind’s ability to categorize aromas is limited to 7 ± 2 major or primary types [42–45]. Examples of these primary aroma categories are presented in Table 1.

The American Society for Testing and Materials (ASTM) has classified 830 aroma descriptors [46]. Nevertheless, human panel tests have indicated that human subjects can correctly identify an average of less than 100 aromas after good training when discriminating familiar aromas [47]. Aromas can be divided into simple and complex categories. A simple aroma is a single, well-defined compound, in most cases previously synthesized, which is considered the main or principal constituent (or with greatest impact) within the aroma of many natural materials. Natural materials, particularly of plant origin, may emit an aroma composed of tens, hundreds, or even thousands of separate chemical species. Moreover, the same aroma can change over time if environmental conditions change, because of effects on volatility. Many plant species also produce very characteristic chemicals called essential oils composed of simple or complex mixtures of compounds that impart a very diagnostic smell attributable only to specific plant species or taxa.

The different volatilities of the molecular species that compose the aroma bouquets are given major consideration in product development within the cosmetics and perfume industries. The most volatile compounds represent the “top notes”, which produce an immediate olfactory impact, whereas the “base notes” are more persistent and subtle aromas usually due to being less volatile at room temperature. These two components characterize the fundamental aroma structure of a perfume or cologne with a particular scent [48]. Hydrocarbons usually do not exhibit odors of interest or of a well-defined character, although certain unsaturated hydrocarbons such as cyclic alkenes have been identified and associated with typical and pleasant notes, such as fruity, green, and floral odors [49].

The search for attractive or pleasing aromas is a key preoccupation in the food industry as well. The characteristics and qualities of a complex aroma, composed of a widely diverse mixture of constituents that collectively produce the unique olfaction sensation that defines a specific product, are key attributes receiving the greatest attention in product-development research. In the case of coffee, over 800 different compounds have been identified as playing a role in determining the coffee aroma [50], of which 35 are considered the most potent aroma elements [51].

### 2.2. Biological Olfaction

Hartman [52], defined flavor as the combined effect of olfactory (aroma) and gustatory (taste) sensations experienced when food is placed in the oral cavity and masticated. More recent studies have added the trigeminal sense to taste and smell in the perception of flavor [53,54]. Gustation receptors located on the tongue respond to four different characteristics of taste – sweet, sour, salt and bitter – when they come into direct contact with soluble compounds. Receptors for the trigeminal sense are located in the soft tissues at the back of the throat, as well as in other parts of the body, and respond to chemically-irritating substances such as those found in hot and spicy foods. Thus, the pungency of ammonia and the coolness of menthol are trigeminal sensations [21].

By the identification of a large family of G-protein-coupled receptors and the invention of advanced molecular and physiological techniques, a detailed description of the mechanisms responsible for stimulus-induced signaling of the olfactory system are now known [35]. The olfactory sensory neurons are located on a specialized membrane (olfactory epithelium) which lines the dorsal aspect of the nasal cavity [55]. These can discriminate between thousands of low molecular mass, mostly organic compounds, representing aliphatic and aromatic molecules with varied carbon backbones and a diversity of functional groups. These are stimulated solely by volatile compounds that reach these receptors by air passing through the nose or the mouth and nasopharynx into the nose.

Sensitivity to aromas can be improved and varies considerably from person to person. Gilbert and Wysosky [56] tested the sensitivity of about 1,500,000 people to selected aromas and discovered that sensitivity varies widely with the nature of the aroma, sex, age, physiological moment and health of the people tested.

Aroma and taste perception also can be affected by some illnesses. Some psychophysical studies have clearly demonstrated the existence of specific anosmias (lack of olfaction or absence of ability to smell), hyposmia (decrease of ability to smell), and parosmia (distorted sense of olfaction, resulting in phantom, non-existent and mostly unpleasant smells) [57–59]. In common with other human sensory perception, the perceived intensity of an aroma is not linearly related to its concentration. High aroma detection thresholds are observed for most aromas that are gases under standard temperature and pressure (STP) conditions, whereas aromas with low vapor pressures generally have low aroma detection thresholds [60].

Many researchers have tried to understand olfactory sensitivity based on specific structural and stereochemical properties of aromatic compounds. Amoore [61] and Ohloff *et al.* [44,45] proposed a relationship between the shape of the molecule, such as the presence of certain functional groups, and features of certain olfactory receptors that respond primarily to compounds with specific three-dimensional molecular profiles elucidated in related studies [62,63]. Other scientists empirically have correlated trends in human aroma detection thresholds with macroscopic properties of aromatic compounds, such as the boiling point of the liquid phase [64].

Typically, there is a sigmoidal relationship between concentration and sensitivity to all aromatic compounds with a lower threshold below which the aroma is not detected and an upper concentration limit above which the perception of aroma intensity levels off. The value of the threshold varies from aroma to aroma and between individuals, as does the midpoint of the curve. Moreover, the perception of aroma intensity grows slowly with increasing concentration [21].

The human thresholds for detection of some strong common odorants, such as citral (lemon) and butyric acid in air, can be quite low (Table 2). This threshold for detection in humans is the minimum concentration (mg dm^−3^), usually in air, at which a human subject can detect a difference in smell between a particular substance in the air compared with a control substance. Similar detection thresholds for taste or flavor are determined with flavor components dissolved in water. The threshold for recognition, the minimum concentration at which the subject can correctly identify the aroma qualities of the compound, is usually higher and varies from person to person according to level of training [21]. The perception threshold for aromas decreases upon extended contact with an aroma. After continued exposure, the perceived intensity falls exponentially, but by removal of the aroma, the intensity recovers [21].

## 3. Conceptual Development of the Electronic Nose

The first studies involving aroma measurements were done in the 1920s by Zwaardemaker and Hogewind [65] who focused on measuring the electricity of a fine spray of water. They found the addition of volatile substances to the water increased the spray-electricity that could be used to detect “the presence of small amounts of aromatic compounds by means other than through the sense of smell.” The first real tool for measuring aromas was developed by Hartman [52] in 1954. The sensing element was a microelectrode, a simple platinum wire of 0.8 mm in diameter, which measured the flow of current by a sensitive millivoltmeter. Hartman also was the first to propose the idea that the apparatus could operate with several different coated sensitive elements, and that some different electrode-coating substances could be capable of giving differential responses with different compounds [66].

Moncrieff [67] worked on the concept that different coatings materials, such as polyvinyl chloride, gelatin, and vegetable fats could be capable of providing different and complementary data for the discrimination of simple and complex aromas. His studies were limited to the use of a single temperature-sensitive resistor, but postulated that an array with six thermistors, provided with six different coatings, could discriminate large numbers of different aromas. In 1965, two other groups published studies and experiments on olfaction devices: Buck *et al*. [68] studied the modulation of conductivity as an answer to differentiating aromas bouquets, while Dravnieks and Trotter [69] used the modulation of contact potential to monitor aromas. These studies have been considered only a first approach to aromas evaluation because of the lack of analytical instruments. However, about 20 years later (1982), the idea of an electronic-nose instrument with an intelligent, chemical array sensor system for aroma classification resulted from studies of Persaud and Dodd [1] and Ikegami and Kaneyasu [70]. By that time, the development of computers and electronic sensors made it conceptually possible to obtain an electronic device capable of imitating the mammalian olfactory system.

The term “electronic nose” was coined in 1988 by Gardner and Bartlett, who later defined it as *“an instrument which comprises an array of electronic chemical sensors with partial specificity and appropriate pattern recognition system, capable of recognizing simple or complex odors”* [71]. In 1991, scientific interest in the use and applications of electronic noses was sanctioned by the first advanced workshop on chemosensory information processing during a session of the North Atlantic Treaty Organization (NATO) that was entirely dedicated to the topic of artificial olfaction. Since 1991, interest in biological sensors technology has grown dramatically as is evident by numerous scientific articles on the subject and commercial efforts to develop and improve sensor technologies and tools of greater sophistication and improved capabilities, diverse sensitivities and with ever-expanding applications.

### 3.1. Electronic Aroma-Detection Sensor Types

The sensor array in an electronic nose performs very similar functions to the olfactory nerves in the human olfactory system. Thus, the sensor array may be considered the heart and most important component of the electronic nose. The instrument is completed by interfacing with the computer central processing unit (CPU), recognition library and recognition software that serve as the brain to process input data from the sensor array for subsequent data analysis.

A good sensor should fulfill a number of criteria. First, the sensor should have highest sensitivity to the target group of chemical compound(s) intended for detection and with a threshold of detection similar to that of the human nose, down to about 10^−12^ g mL^−1^ [72]. To be most useful with diverse detection capabilities, e-nose sensors should have relatively low selectivity in order to be sensitive to a wide number of different chemical compounds. The electronic nose sometimes is used in quality assurance-quality control (QA-QC) applications to maintain controlled laboratory conditions or to assure product uniformity in manufacturing. More often e-noses are used in uncontrolled environmental conditions in the open-air for field applications. For these applications, the sensor array must have low sensitivity to variable environmental parameters, in particular to temperature and air humidity. Sensors should be capable of operating at relatively low temperatures when necessary, have short calibration and training requirements, fast recovery time between runs and maintenance procedures to maintain low operating costs. They must also have short recording and analysis times, particularly when used as on-line systems, and high sensor array stability. As most applied markets and industries tend to move more toward miniaturization of analytical instrumentation, the sensor array must ultimately be very portable and small for convenient diverse operations and with built-in recording and analysis capabilities.

The basis of electrochemical gas sensor operation involves interactions between gaseous molecules and sensor-coating materials which modulate electrical current passing through the sensor, detectable by a transducer that converts the modulation into a recordable electronic signal [21]. There are many different types of electrochemical sensors (e.g. metal-oxide gas sensors, metal-oxide semiconductor field effect transistors, conducting polymer gas sensors, acoustic wave gas sensors, quartz crystal microbalance sensors, surface acoustic wave devices, field-effect gas sensors, electrochemical gas sensors, pellistors, fiber-optic gas sensors) and many different types of sensor-coating materials which are classified according to additive doping materials, the type and nature of the chemical interactions, the reversibility of the chemical reactions and running temperature. A summary of the types and mechanisms involved with some common gas sensor technologies are listed in Table 3.

Transducer recording devices of various types in electronic-nose sensors are categorized according to the nature of the physical signal they measure. The most common methods utilize transduction principles based on electrical measurements, including changes in current, voltage, resistance or impedance, electrical fields and oscillation frequency. Others involve measurements of mass changes, temperature changes or heat generation. Optical sensors measure the modulation of light properties or characteristics such as changes in light absorbance, polarization, fluorescence, optical layer thickness, color or wavelength (colorimetric) and other optical properties.

The most widely used class of gas sensors are the metal-oxide gas sensors. They were first used commercially in the 1960s as household gas alarms in Japan [72]. More recent uses include applications in many different industrial processes. Basically, a metal-oxide sensor consists of a ceramic support tube containing a heater spiral, usually composed of platinum. The most widely used coating material is tin-dioxide (SnO_2_), doped with small amounts of catalytic metal additives (also called Taguchi sensors). The sorption of gas molecules provoke changes in conductivity brought about by combustion reactions with oxygen species on the surface of the tin-dioxide particles. These sensors by necessity operate at high temperatures ranging from about 300 °C to 550 °C. At lower temperatures, the rate of the reactions on the oxide surface is too slow. At temperatures below 100 °C, the low vapor pressure of water molecules inhibits oxidative chemical reactions [73]. The consequence of this high operating temperature is very high power consumption.

Metal-oxide sensors have very high sensitivity (sub-ppm levels for some gases) and respond to oxidizing compounds (zinc-oxide, tin-dioxide, titanium-dioxide, iron oxide) and some reducing compounds, mainly nickel-oxide or cobalt-oxide [74]. From a chemical point of view, the sensing reaction is based on an oxygen exchange between the volatile gas molecules and the metal coating material. Electrons are attracted to the loaded oxygen and result in decreases in sensor conductivity [75].

The metal-oxide semiconductor field effect transistors (MOSFET) were firstly reported by Lundström *et al.* [76] in 1975 based on the tendency of a number of metals to adsorb and dissolve hydrogen [77]. The first hydrogen-sensitive MOSFET used palladium film as gate electrodes [76]. Since then, other combinations of metal oxides and catalysts such as palladium-titanium dioxide [78], palladium-gallium arsenide [79], and palladium-zinc oxide [80] have been investigated. Metal oxide semiconductor (MOS) sensors consist of three layers: a silicon semiconductor, a silicon oxide insulator and a catalytic metal through which the applied voltage creates an electric field. When polar compounds interact with the metal, the electric field is modulated and recorded by the transistor [72]. The doping metal (or gate) can be a thick (100–200 nm) or thin (6–20 nm) film. In the first case, the sensor can only respond to dissociated hydrogen. Thus, sensor sensitivity to hydrogen non-releasing molecules such as ammonia or carbon monoxide is very low. A thinner layer of metal on the sensor changes the catalytic activity towards these kinds of molecules [81–83].

Conducting or conductive polymer gas sensors operate based on changes in electrical resistance caused by adsorption of gases onto the sensor surface. Conductive electroactive polymers have attracted much interest for use as electronic noses since the early 1980s [84], particularly because they have high sensitivities, short response times, are easily synthesized, have good mechanical properties and are particularly useful because they operate at room temperature [85]. Conductive polymer gas sensors consist of a substrate, usually silicon, a pair of gold-plated electrodes and a conducting organic polymer coating as the sensing element [72]. The sensitivity of conductive polymers to VOCs is measured as changes in electrical resistance. Conducting polymers are usually synthesized by chemical or electrochemical oxidizing of the corresponding monomers. The most widely used sensor coating monomers are polypyrrole, polyaniline and polythiophene [21], but polyacetylene, poly(phenyl vinylene), poly(3,4-ethylenedioxythiophene), poly(*N*-vinylcarbazone), poly(thienylenevinylene) and many others have been investigated [39,85]. The common feature of conductive polymer materials is the presence of a conjugated pi-electron system which extends over the whole polymer.

One of the main weaknesses of conductive polymers is their high susceptibility to ambient environmental humidity, although the sorption of water within polymer films may play an important role in the mechanism of gas sensitivity [86]. The sensitivities of conducting polymer films are generally an order of magnitude lower than metal oxide films; nevertheless, measurements at the ppm and sub-ppm level have been reported for some analytes with suitable electronic circuitry [87].

Acoustic wave gas sensors use a mechanical (acoustic) wave as the sensing mechanism. As the acoustic wave propagates through or on the surface of the sensor coating material, any changes to the characteristics of the propagation path, due to the sorption of VOCs, affect the velocity and/or amplitude of the wave [88,89]. They consist of a piezoelectric substrate, usually quartz (SiO_2_), lithium niobate (LiNbO_3_), lithium tantalite (LiTaO_3_) or zinc oxide, doped with a suitable sorptive material.

The first report of an acoustic wave chemical vapor sensor was in 1979 [90], even though acoustic wave devices had been in commercial use for over 60 years – mainly in the telecommunications industry. These devices are small, inexpensive and sensitive to virtually all gases, particularly in proportion to the mass of adsorbed compounds whose molecular weight is one of the fundamental parameters for analyte identification [91]. Two main types of acoustic wave gas sensors have been developed: 1) those based on bulk acoustic wave (BAW) devices and 2) those based on surface acoustic waves (SAW) devices. In BAW devices, the wave propagates through the substrate. With SAW devices, the wave propagates on the surface of the substrate [88]. In both cases, the waves are at ultrasonic frequencies, typically 1 to 500 MHz [21].

Acoustic wave sensor sensitivity to VOCs is determined by the types of sorptive coatings used on the sensors. Different materials have been used for this purpose: monolayer films [92], surface-attached molecules [93], and layers of a wide variety of polymeric films [94–96].

The thickness shear mode resonator (TSM), also referred to as a quartz microbalance (QMB), is the best-known, oldest and simplest type of piezoelectric acoustic wave device [88]. It comprises a slice of single crystal quartz, typically around 1 cm in diameter, with metal electrodes, usually gold, evaporated onto the two large faces connected to lead wires [97]. The sensor is typically used in an oscillator circuit. Upon excitation by application of a suitable voltage across the two electrodes, a reduction in the oscillation velocity indicates mass accumulation on the sensor surface. Typically, the resonance frequency of the device is in the range of 5 to 20 MHz (Scarpa, pers. comm.).

Electrochemical gas (EC) sensors operate at room temperature, have low power consumption and are very robust, but still quite bulky [21]. Their sensing methodology is based on the electrochemical oxidation or reduction of volatile molecules at a catalytic electrode surface [98]. This technology has a good relevance when applied to the detection and measurement of electrochemically active gases, but they are not very sensitive to a wide diversity of compounds, especially aromatic hydrocarbons [39]. The sensitive electrode typically is composed of a layer of precious metal, such as gold combined with carbon, coated onto a hydrophobic membrane. Commercial electrochemical gas sensors are currently available for a wide range of toxic gases (Table 4). In each case, the concentration of gas is determined by measuring the current flowing in the sensors. They are commonly used in mining operations for personnel-protection monitoring, tunneling and other industrial applications. The disadvantages of these sensors, from the standpoint of their application within electronic nose, are their size and relatively high selectivity for a limited number of simple gases [99].

The calorimetric or catalytic bead (CB) sensor consists of two coils of fine platinum wire embedded in a bead of alumina connected in a Wheatstone bridge circuit. One of the pellistors is impregnated with a special catalyst that promotes oxidation. The other pellistor is treated to inhibit oxidation. Current is passed through the coils to heat the bead until oxidation of the sample gas occurs at 500–550 °C. Combustion of the gas raises the temperature further and increases the resistance of the platinum coil in the catalyzed bead, leading to an imbalance of the bridge. This output change in resistance is linear for most gases and response time is a few seconds. The sample gas must contain at least 12% oxygen by volume for oxidation. The Area REA monitor produced by Rae Systems is an example of combined-technology e-nose that contains a CB sensor.

Optical sensor systems are somewhat more complex than typical sensor-array systems having transduction mechanisms based on changes in electrical resistance. Optical sensors work by means of light modulation measurements and consist of an assortment of technologies ranging from diverse light sources with optical fibers to various photodiode and light-sensitive photodetectors. Various operational modes have been developed that measure changes in absorbance, fluorescence, light polarization, optical layer thickness, or colorimetric dye response. The simplest optic sensors use color- changing indicators, such as metalloporphyrins, to measure absorbance with a LED and photodetector system upon exposure to gas analytes. Two specialized types of optical sensors are the colorimetric and fluorescence sensors. Colorimetric sensors use thin films of chemically-responsive dyes as a colorimetric sensor array. Fluorescence sensors detect fluorescent light emissions from the gas analyte at a lower wavelength and are more sensitive than colorimetric sensor arrays.

There are a variety of advantages and disadvantages of using various e-nose sensors based on their response and recovery times, sensitivities, detection range, operating limitations, physical size, inactivation by certain poisoning agents, and other limitations that are specific to individual sensor types. The types and categories of advantages and limitations associated with individual e-nose sensor types are closely linked with the nature of the technology that determines the principle for detection and the types of gas analytes that may be detected with each sensor type. A listing of some of the major advantages and disadvantages associated with each e-nose sensor type are summarized in Table 5.

Thus, the unique combinations of advantages and disadvantages related to individual sensor types largely determines the range of capabilities and potential applications that each sensor type provides for the analysis of various gas analytes in specific operating situations. Some other important considerations for sensor selection include operational expenses, maintenance costs, training costs and ease of use by the operator.

Conducting polymer and electrochemical sensors are probably the most versatile e-nose sensor types available due to operation at ambient or room temperature, low power consumption, good sensitivity to a wide range of gas or volatile analytes, and inexpensive operating costs. Conducting polymers are available in a very large diverse range of sensor coating types providing almost unlimited combinations of sensors in the array for analysis of any specific organic chemical classes or VOC mixture types possible in any particular application. This versatility of conducting polymer sensors is especially true as the number of sensors in the array increases although more sensors is not necessarily better for efficiency of detection, portability, or operating costs. Electrochemical sensors are somewhat more limited than conducting polymers due to their bulky size and limited sensitivities to simple gases. By contrast, metal oxide and calorimetric or catalytic bead sensors must operate at high temperatures, resulting in greater operating costs, and have much more limited range of detectable analytes. Nevertheless, certain analytes require high-temperature sensors for effective detection and sensitivity.

## 4. Electronic Nose Instrumentation

Gardner and Bartlett [71] provided a basic requisite definition of an electronic-nose device with a list of necessary components (as follows):

1. an aroma delivery system, which transfers the volatile aromatic molecules from the source material to the sensor array system
2. a chamber where sensors are housed: this has usually fixed temperature and humidity, which otherwise would affect the aroma molecules adsorption
3. an electronic transistor which converts the chemical signal into an electrical signal, amplifies and conditions it
4. a digital converter that converts the signal from electrical (analog) to digital
5. a computer microprocessor which reads the digital signal and displays the output after which the statistical analysis for sample classification or recognition is done.

It is inferable from the Gardner-Bartlett definition that for a detection devise to be considered an electronic nose it must contain an intelligent chemical-array sensor system that mimics the mammalian olfactory system and is used specifically to sense aromatic VOCs. The implication is that all sensing devices that have only one sensor or can detect only one compound or aroma (electronic aroma monitors) cannot by definition be considered electronic noses. Thus, electrochemical cells (ECs) that detect only one specific gas are not electronic noses according to the Garner-Bartlett definition.

The typical complete sampling time for e-nose analyses is a function of the sensor material, the aroma elements being analyzed, the operating temperature of the sensor, the ambient humidity, the statistical method used to analyze the results, and the accuracy of the microprocessor. Generally, a rise-time of 30 s is observed from a MOS sensor at 350 °C, and 10 s for a conducting polymer sensor at room temperature [21].

The aroma delivery system together with the sensor array system is the most important part of the electronic nose device because volatile compound adsorption or contact with the sensor surface is *conditio sine qua non* sensing. The simplest possible aroma delivery system is manual headspace sampling. The aromatic material is stored in a closed volume and allowed to build headspace. The volatile compounds are removed from the sample vessel using a syringe and injected into the sensor chamber maintained at a constant temperature and purged with a clean reference gas (usually conditioned or filtered environmental air) after sensor readings. The automatic headspace delivery system can significantly reduce the sampling time and standardize the aroma concentration. The flow-injection method employs a carrier gas (clean environmental air, CO_2_ or N_2_) which leads the headspace volatiles to the sensor chamber maintained at controlled humidity and temperature for precision. The main drawbacks of flow-injection methods are the high cost and slow response time [21].

Many electronic noses are commercially available today and have a wide range of applications in various markets and industries ranging from food processing, industrial manufacturing, quality control, environmental protection, security, safety and military applications to various pharmaceutical, medical, microbiological and diagnostic applications. A summary of some of the most widely used electronic noses with manufacturers, models available and technological basis are listed in Table 6. These represent a wide diversity of sensor types based on uniquely different technologies. The list includes instruments with single-technology sensor arrays and combined-technology instruments that consist of e-noses working in tandem with classical analytical systems. The additional need to identify individual chemical species or components within sample mixtures, beyond the identity of the sample (source) as a whole, recently has caused a necessary merger of electronic nose technologies with purely analytical instruments. These technological mergers have resulted in new instrumentations that have confused the border between electronic noses and conventional analytical instruments. The blurring of instrument nomenclature for these new hybrid devices has created a challenge for manufacturers and scientists who are experiencing increasing difficulty in naming these hybrids with consistent nomenclature due to the absence of standardized naming conventions. Nevertheless, the division between pure electronic-nose instruments based on collective sensor-array outputs and classical analytical instruments with single-detector outputs are fairly unequivocal. Classical analytical instruments and detectors such as electron capture detectors (ECD), flame ionization detectors (FID), flame photometry detector (FPD), gas chromatographs (GC), infrared spectrometers (IRS), ion mobility spectrometers (IMS), mass spectrometers (MS), nuclear magnetic resonance spectrometers (NMRS), photo-ionization detector (PID) and quadrupole fingerprint mass spectrometers (QFMS) are not considered e-noses in the strictest sense because they do not provide a collective data output from a sensor array and are designed to detect and identify individual components of a gas mixture.

The uses of electronic noses have grown rapidly as new applications have been discovered. The numbers of e-noses sold by various manufacturers has largely depended on the technology basis of individual instruments, costs per unit, and specific application needs [100]. In 1997, there were about 500 total desk-top analytical instruments units sold worldwide with an approximate market value of $30 million euros [21]. Within the past ten years, the Applied Sensor Company has sold the most units (> 100,000) of their e-nose (the Air Quality Module electronic nose); primarily used to maintain ambient or environmental air quality by detection of odors, VOCs and carbon dioxide within living spaces [101]. Since the late 1990s, most scientific research done hitherto to evaluate and compare the sensing capabilities and applications of electronic noses has focused on relatively few prototypes that are the basis of the following discussion.

The Alpha-MOS (Toulouse, France) Fox electronic nose was designed in collaboration with the Universities of Warwich and Southampton. It employs either six (Fox 2000), 12 (Fox 3000) or 18 (Fox 4000) metal oxide gas sensors and can be used with external carrier gas bottles in a flow-injection system, or with an internal pump and mass-flow controller. The Aromascan A32S (Osmetech Plc, UK) is an organic matrix-coated polymer-type 32-detector e-nose based on an earlier design using technology arising from the University of Manchester Institute of Science and Technology. This instrument is no longer commercially available because Osmetech Plc discontinued production and redirected their business toward development and production of instruments for predominantly biomedical applications. The conducting (or conductive) polymers used to coat the sensors in the array were produced by electropolymerization of either polypyrrole, polyanaline or polythiophene derivatives that were modified with ring-substitutions using different functional groups that impart unique conductive properties [102].

The Cyranose 320 (Cyrano Science, Pasadena, CA, USA) is a portable electronic-nose system whose component technology consists of 32 individual polymer sensors blended with carbon black composite and configured as an array [103]. Airsense PEN2 and PEN3 (Airsense Analytics GmbH, Schwerin, Germany) e-noses contain a very small and portable 10 metal-oxide semiconductor (MOS) gas sensor array with a small-volume measuring chamber. It can be linked with an adsorbent trapping unit or a headspace auto sampler for laboratory analyses. The quartz microbalance technology employed in the LibraNose 2.1 sensor array (Technobiochip, Pozzuoli, NA) uses eight 20 MHz AT-cut quartz microbalance (QMB) sensors with a gold surface (Gambetti Kenologia, Binasco, PV, Italy) coated with either metalloporphyrines, deposited by solvent casting, or by polypyrrole polymer films. The coating films are deposited by means of Langmuir-Blodgett casting technology utilized at the Technobiochip thin-film deposition unit.

## 5. Data Analysis for Electronic Noses

The digital outputs generated by e-nose sensors have to be analyzed and interpreted in order to provide useful information to the operator. Commercially available analysis techniques fall into three main categories as follows [72]:

1. Graphical analyses: bar chart, profile, polar and offset polar plots
2. Multivariate data analyses (MDA): principal component analysis (PCA), canonical discriminate analysis (CDA), featured within (FW) and cluster analysis (CA)
3. Network analyses: artificial neural network (ANN) and radial basis function (RBF)

The choice of method utilized depends on the type of available input data acquired from the sensors and the type of information that is sought. The simplest form of data reduction is graphical analysis useful for comparing samples or comparing aroma identification elements of unknown analytes relative to those of known sources in reference libraries. Multivariate data analysis comprises a set of techniques for the analysis of data sets with more than one variable by reducing high dimensionality in a multivariate problem when variables are partly correlated, so they can be displayed in two or three dimensions. For electronic-nose data analysis, MDA is very useful when sensors have partial-coverage sensitivities to individual compounds present in the sample mixture. Multivariate analysis can be divided into untrained or trained techniques. Untrained techniques are used when a database of known samples has not been previously built, therefore it is not necessary nor intended for recognizing the sample itself, but for making comparisons between different unknown samples to discriminate them. The simplest and most widely used untrained MDA technique is principal component analysis. PCA is most useful when no known sample is available, or when hidden relationships between samples or variables are suspected. On the contrary, trained or supervised learning techniques classify unknown samples on the basis of characteristics of known samples or sets of samples with known properties that are usually maintained in a reference library that is accessed during analysis.

The artificial neural network (ANN) in the best known and most evolved analysis techniques utilized in statistical software packages for commercially-available electronic noses. Mimicking the cognitive processes of the human brain, it contains interconnected data processing algorithms that work in parallel. Various instrument-training methods are employed through pattern-recognition algorithms that look for similarities and differences between identification elements of known aroma patterns found in an analyte-specific reference library. The training process requires a discrete amount of known sample data to train the system and is very efficient in comparing unknown samples to known references [104]. The result of ANN data analysis usually is in the form of a percentage match of identification elements in the sample with those of aroma patterns from known sources in the reference library.

## 6. Electronic-Nose Applications

Electronic-nose systems have been designed specifically to be used for numerous applications in many different industrial production processes. A wide variety of industries based on specific product types and categories, such as the automobile, food, packaging, cosmetic, drug, analytical chemistry and biomedical industries utilize e-noses for a broad and diverse range of applications including quality control of raw and manufactured products, process design, freshness and maturity (ripeness) monitoring, shelf-life investigations, authenticity assessments of premium products, classification of scents and perfumes, microbial pathogen detection and environmental assessment studies (Table 7). Some individual examples of electronic nose applications in each of these individual industries and product areas are discussed in more detail in the following sections.

### 6.1. Food Freshness, Quality, Ripeness and Shelf-Life

The age of fruits (ripeness or maturity level) determines the shelf life and future rate of quality loss due to changes in flavor, firmness and color. Harvesting fruits at an optimal physiological condition ensures good quality at a later stage (when evaluated by the consumer) by enhancing a number of quality characteristics that extend the shelf-life, slow the rate of decline in firmness or texture, and maintain a preferred level of flavor and overall appearance.

Currently, traditional measuring techniques such as the starch conversion index and flesh firmness or pressure test are used to determine fruit quality. These testing methods are destructive and involve random sampling to assess fruit quality. Consequently, individual fruits or fruit clusters are not graded for quality assessments needed for optimizing treatments and marketing strategies. Thus, there is a need for non-destructive techniques to assess fruit quality based on aroma characteristics that are highly correlated with all of the factors that affect shelf-life and future marketability. Shaller *et al.* [72] provided a very good review of electronic-nose systems that have been used for various applications with different types of foods.

Several studies have demonstrated that the aroma emitted by fruits can indicate the maturity level and thus quality and shelf-life of the marketed product. Pathange *et al.* [105] used maturity indices such as starch index and puncture strength to categorize fruit of the “Gala” apple variety into three maturity groups referred to as immature, mature and over-mature fruits. Multivariate analysis of variance of the e-nose sensor data indicated that the instrument could classify the fruit into the correct group in 87% of the samples. Oshita *et al*. [106] examined the shelf-life of “La France” pears that were grouped into three storage treatments after being harvested and judged using e-nose data as either immature, mature or over-mature. The aromas of pears from the three treatments were classified into three classes based on their physiological states determined from distinct aroma patterns derived from a 32-sensor array output.

Gòmez *et al*. [107] studied volatile production of unripe, half-ripe, full-ripe and over-ripe tomatoes using the PEN 2 E-nose (10 different metal oxide sensors) with principal component analysis (PCA) and linear discriminant analysis (LDA). The results demonstrated that the electronic nose could differentiate among the ripeness states of tomatoes and classify them with 100% reliability in each ripeness group. They also evaluated the same e-nose for its capacity to monitor changes in volatile production of mandarin oranges during different storage treatments. In this case, the storage shelf-life was better distinguished using LDA than PCA. However, the correlation between measured and predicted values of fruit quality showed reasonably good predictive performance using unanalyzed sensor outputs.

The process of coffee production has been widely investigated by e-nose technologies to distinguish different types of coffee beans [25], to identify various brands and mixtures [108], to classify commercial coffee blends [109] and separate samples with different roasting levels [110]. Falasconi *et al.* [111] provided evidence for the efficacy of predicting the perfect ripening moment by analyzing roasted coffee with a new thin film semiconductor metal oxide gas sensor e-nose (Electronic Olfactory System EOS835). The human panel expert-taster assessment indicated that the best coffee quality resulted when samples lots were ripened for 96-h. The electronic nose effectively recognized ripening progression and allowed the determination of necessary coffee quantity and operating conditions needed to detect samples at the perfect 96-h ripening times.

Other studies involved in predictions of fruit maturity level and shelf life have been done on various fruits. For example, fried mango chips were evaluated for the presence of deteriorative aromas [112]. Fuji apples were evaluated for the effects of different storage conditions, storage periods and days of shelf-life on ripening and condition [113]. Supriyadi *et al*. [114] investigated the specific aroma of a pentane extract in snake fruit. Others have examined the pre- and post-harvest characteristics of kiwifruit [115], and fresh-cut vegetables like chicory [116].

Utilizing e-noses as a means of monitoring fruit freshness and shelf-life prior to marketing can have a number of benefits that maximize corporate profits and optimize customer satisfaction. Information from e-noses on fruit physiological states, based on changes in released volatiles, can be applied to retard the ripening process through exposure of the fruit to ripening inhibitors (such as cyclopropene compounds that act as ethylene-receptor blockers) at the appropriate time, adjustments in fruit storage conditions to preclude ethylene accumulation (most associated with fruit ripening), and removal of bruised or damaged fruits that enhance ripening of surrounding fruits and contribute to storage losses due to rots, decays, and various fruit diseases.

### 6.2. Milk and Dairy Products

Dairy products contain off-flavor compounds created by a variety of mechanisms such as through the action of natural and microbial enzymes and chemical changes catalyzed by light or heavy metals. In cheeses, quality, flavor and taste are closely connected to the ripening process which depends on the growth of bacteria, lipid degradation and oxidation, and proteolysis. Traditionally, sensory analysis was used to determine the product identity of cheese. However, detection of aroma compounds using electronic noses has become more and more important.

Russell [117] first focused on the classification of Parmesan cheeses with differing rates of maturity. The Aromascan e-nose successfully distinguished the two types. They also were able to characterized Gorgonzola and Cottage cheese using polypyrrole semi-conductor sensors. Zondevan *et al.* [118] used the electronic nose to classify block milk products, subjected to various heating processes, in order to predict the most favorable heating method. In this case, the results were moderate to good. Ampuero *et al*. [119] found the electronic nose has a lower detection limit (0.5 mg/kg) and better precision compared to dynamic headspace gas chromatography (GC) for determining the presence of trimethylamine in Swedish milk samples.

Compared with near-infrared spectroscopy (NIRS), the electronic nose has shown better results. Riva *et al.* [120] demonstrated that NIR spectra (NIRS) collected from Crescenza cheese were influenced both by raw material and by its high moisture content. NIRS, like the classical measures such as acidity and texture chances, was able to detect only chemical modifications that occurred during the first stages of storage, whereas the results obtained by spectroscopy and electronic nose analyses were more useful because they could be used to monitor the shelf-life of these products as on-line sensors.

The shelf-life of milk also has been studied [121]. A Fox 4000 electronic nose equipped with 18 sensors and an autosampler was used to evaluate the growth of total bacteria in milk stored at ambient temperature and 5 °C. The results showed that measurements generated by the electronic nose could be used to detect both bacterial growth in milk and shelf-life. Other studies that have focused on the feasibility of the electronic nose in evaluating the shelf-life of several other dairy products including yogurts [122], Taleggio cheese [123], Danish Blue Cheese [124]; seasonal changes in whole milk powder aromas [125], and the selection of bacterial strains of *Lactobacillus casei* as flavor-producing adjunct cultures [126].

### 6.3. Meat Products

Much work has been done in the electronic detection of quality characteristics of meat products within the food industry. Berdagué and Talou [127] studied instruments based on MOS sensors, starting in 1993 with the Alabaster-UV as one of the first commercially available e-nose instruments. This system consisted of a stainless steel measurement chamber containing one semiconductor gas sensor, a UV-lamp, air inputs, and outputs connected to a fan. The signal obtained was displayed as deadsorption curves in “Alabaster” units. It was shown that this simple instrument could differentiate maxima in aroma perception resulting from the maturation of dry non-spiced meats, and rapidly detected sex-linked differences in meat product composition.

Vernat-Rossi *et al*. [128] demonstrated that non-controlled ambient air that simulated on-line quality control could be used in the rapid discrimination of food products. This research was carried out on sausages, and the data obtained was subjected to modeling by the Gompertz sigmoidal function and discriminant analysis with backward variable selection. In both cases, the results were satisfactory with 100% and 97% product recognition, respectively.

Rajamäki *et al.* [129] studied the applicability of an electronic nose for the quality control of modified-atmosphere packaged broiler chicken cuts with different temperature regimes. The electronic nose results were compared with those obtained by microbiological, sensory, and headspace GC analyses. The e-nose could clearly distinguish broiler chicken packages with deterioration from fresh packages either earlier or at the same time that sensory changes indicated significant deterioration. Counts of *Enterobacteriaceae* and hydrogen sulphide-producing bacteria were most consistently associated with the electronic nose results indicating that the electronic nose was capable of detecting even early signs of spoilage in modified atmosphere packed poultry meat.

Vestergaard *et al.* [130] found the storage time of a pork-meat pizza topping product was predictable using an electronic nose. The study included two independent test sets composed of “known” production samples and “unknown” production samples. The results showed that storage time of “known” samples was very well predicted, while the “unknown” storage time was fairly well predicted. This provided evidence that the electronic nose system was a relevant and useful device for on-line implementation in quality control of pork meat products.

### 6.4. Fish and Seafood Products

Ólafsson *et al.* [131] first evaluated spoilage in three different fish species (haddock, cod and redfish) using two to six MOS sensors. The samples were kept at room temperature or on ice, and the results compared with sensory analyses. The results were promising enough to prompt further investigation into the use of electronic noses in fish freshness assessment. In fact, Winquist *et al*. [132] trained a system based on MOSFET sensors to predict the age of cod filet. This study showed some very convincing results on the feasibility of the system.

Jonsdottir *et al.* [133] attempted to standardize cod-roe products by using an electronic nose. Flavor profiles of commercially-processed ripened roe from Iceland and Norway were studied by sensory analysis, gas chromatography-olfactometry, gas chromatography-mass spectrometry, and an electronic nose to characterize the headspace of ripened roe. The analysis confirmed the presence of aroma compounds contributing to the typical ripening and spoilage flavors detected by the sensory analysis. Two compounds, 3-methyl-1-butanol and 3-methylbutanal, detected with the electronic nose were found to be quality indicators for objectively assessing the ripening of roe.

Olafsdottir *et al*. [134] focused on the feasibility of a prototype gas-sensor array system, the FishNose, built to study the VOCs of fish. In their initial work, they studied the quality changes of cold smoked salmon from four different smokehouses in Europe. Samples were stored in different packaging for up to four weeks under controlled storage conditions at 5 and 10 °C. Quality criteria based on sensory attributes (sweet/sour, off and rancid aroma), and total viable counts and lactic acid bacteria counts were established and used in the classification of samples based on responses of the FishNose. The data outputs from the gas-sensors correlated well with sensory analysis of spoilage aroma and microbial counts, suggesting that they could detect volatile microbial-produced compounds causing spoilage aromas in cold-smoked salmon during storage. The system, therefore, was ideal for fast quality control determinations of freshness evaluation in smoked salmon products.

Haugen *et al.* [135] confirmed the feasibility of the FishNose on direct quality measurements of smoked salmon. Quality changes were monitored by the FishNose and compared with results of traditional sensory, chemical and microbial measurements. In this case, gas-sensor selection was optimized for the detecting of changes in the highly volatile compounds mainly representing microbial metabolism during spoilage. The system was further tested on-site in a smoked salmon production plant. Due to varying ambient air conditions at the production plant during the measurements, the sensor readings had to be corrected by subtracting the background ambient air signal from the sensor readings. High rates of correct sample discrimination were obtained for fresh (95%) and tainted (93%) meat samples, respectively.

Chantarachoti *et al*. [136] evaluated the capability of a portable electronic nose in detecting spoilage of whole Alaskan pink salmon stored at 14 °C and in slush ice. In 92% of the samples, the instrument could correctly classify the fish as either fresh or spoiled.

### 6.5. Agricultural Plant Production

The aroma of grains is the primary criterion of fitness for consumption in many countries. However, the sniffing of grain lots for quality grading is potentially hazardous to humans and should be avoided because of inhalation of toxic or pathogenic mold spores such as from *Aspergillus* species. Jonsson *et al.* [137] used an electronic nose with three different complementary sensors (ten gas sensitive metal-oxide-semiconductor field effect transistors, four tin dioxide-based sensors and a CO_2_ sensor to test samples of oats, rye and barley with different aromas, and wheat with different levels of ergosterol, fungal and bacterial contamination. The ANN could predict the aroma classes of good, moldy, weakly and strongly musty oats with a high degree of accuracy. The ANN also indicated the percentage of moldy barley or rye grains in mixtures with fresh grains. In wheat, a high degree of correlation between ANN predictions and measured ergosterol as well as fungal and bacterial colony forming units (CFUs) was observed.

Di Natale *et al*. [30] used a sensor array formed by five different metal-oxide semiconductor thin-film-based sensors in order to recognize five different vintage years (1989–1993) of the same wine. In those years, the quality and percentage composition of the grapes were almost constant according to the wine-making procedure. The sensors were composed of different materials in order to endow the array with a broad-sensitivity spectrum and to avoid the occurrence of duplication of single-sensor performance. PCA was used for the extraction of pattern features from the sensor data set. Because of the long and special training time necessary for a human to learn to recognize different ages of wine, they wanted to explore the feasibility of an electronic nose that would provide better reproducibility. The results showed that 1990, 1991 and 1992 wines were well separated from the other two, whereas the 1989 and 1993 vintages had aroma bouquets that were totally indistinguishable from one another. This was found to be due to the use of a particular wine-making procedure, the so-called “barricatura” process that imparts a particular aroma to the wine.

Campagnoli *et al.* [138] tested the feasibility of using the electronic nose for detecting processed animal proteins in feedstuffs. The test samples consisted of a compound feed for bovine fortified with processed animal proteins from meat, bone meal, and/or fish meal at different concentrations. The aroma profile was determined by ten metal-oxide semi-conductive sensors. In this study, the electronic nose was able to discriminate the blank sample from all other samples containing processed animal proteins.

The electronic nose also has been used in the field of micropropagation. Komaraiah *et al.* [139] used 19 sensors composed of different metal-oxide semi-conductors and one carbon dioxide sensor in an electronic nose to monitor plant cell cultures. The device was used to continuously monitor the off-gas from two plant cell suspension cultures (*Morinda citrifolia* and *Nicotiana tabacum*), cultivated under batch conditions. By analyzing the multi-array responses using two pattern recognition methods (PCA and ANN), it was possible to monitor the course of the cultivations and, in turn, to predict the biomass concentration in both systems and the formation of a secondary metabolite (antraquinone) produced by *M. citrifolia*.

Scientists working in stirpicultural research have demonstrated that the electronic nose can accelerate the selection of new commercial plant cultivars. Because of the large chemical diversity of oregano (*Origanum vulgare*) subspecies, Bernáth *et al.* [140] selected cultivars for 8 years to obtain desirable lines based on an evaluation of agronomic and essential oil characteristics. Because the aroma of the herb is of commercial importance and highly appreciated by commodity traders as an essential oil, the NST-3320 electronic nose (Applied Sensor, Sweden) was used to discriminate selected lines. This e-nose proved to be an appropriate tool for the identification of cultivars and was potentially useful for accelerating the selection process.

### 6.6. Plant Pathology

The study by Nilsson [141] in 1996 was perhaps the first published attempt to apply an electronic nose technology to plant pathology by discriminating sapwood of Scots pine (*Pinus sylvestris*) colonized and decayed by specific wood decay fungi. Preliminary results indicated that the electronic nose appeared to detect differences, but the sensor array was highly poisoned by moisture in the decayed wood samples which clouded the output and results. The problem of moisture effects on sensory outputs was solved by Wilson *et al.* [142–144] who detected and identified many strains of phytopathogenic microbes (bacteria and fungi) and wood decay fungi, both in pure culture and in wood, using the 32-sensor Aromascan A32S. The overriding background signal caused by sensor responses to the moisture component was eliminated by reducing the relative humidity of the reference air in the sample chamber to 4%. Furthermore, the sensitivity and stability of data output from the sensor array was improved dramatically by using static sampling to avoid sample dilution and air-perturbation effects on sample concentration caused by the continuous flow of reference air into the sampling chamber. This work pioneered the use of e-noses for plant disease diagnosis applicable to all types of microbial plant pests and phytopathogenic microorganisms.

Momol *et al.* [145] presented corroborative evidence of e-nose efficacy using a different type of electronic nose (the e-Nose 4000) to distinguish seven species of plant pathogenic bacteria that were correctly classified with an accuracy of 95% to 100%. Good results also were obtained by Hamilton *et al.* [146]. They used a polypyrrole sensor array system for the detection of *Serpula lacrymans*, a wood destroying dry rot fungus responsible for millions of dollars of damage annually to buildings containing lumber or timbers in Northern and Central Europe, Australia, and Japan. Principal component analysis (PCA) broadly grouped the samples although there was some overlap in the data. Linear discriminant analysis was much more successful, classifying the samples with 0% error. Baietto *et al.* [147] further demonstrated the feasibility of using several types of e-noses for detecting incipient decays in artificially-inoculated wood. Baietto [148] also obtained considerable evidence to support the efficacy of electronic noses as new non-invasive tools for the assessment of decays in live urban trees. This work could eventually lead to a simple and effective means of evaluating the structural integrity of standing trees in the urban environment using e-noses in order to prevent or minimize catastrophic property damage and personal injuries attributed to landscape tree failures.

### 6.7. Plant Identifications

Electronic noses also have been used for the identification of wood samples derived from unknown woody plant sources. Wilson and Lester [149] first reported preliminary results on the usefulness of an e-nose for the identification of woody hosts of forest pathogens based on analysis of volatiles from decayed or diseased wood. Wilson *et al.* [150] demonstrated that the electronic nose easily could be used to identify and classify many different kinds of wood and tree species using extracted wood samples. They recorded electronic aroma signature patterns (EASPs), derived from conductive polymer analyses (CPA) of volatiles from woody cores, to distinguish between 23 tree species from 14 plant families. They also were able to discriminate between oaks (*Quercus* species) in the white oak group (subgenus *Quercus* section *Quercus*) from those in the red/black oak group (subgenus *Quercus* section *Lobatae* Loudon). Many potential applications of this research were recognized in the area of plant ecology and forest management research through chemotaxonomy and the identification of the roles that wood-inhabiting organisms play in stand dynamics and ecosystem functions.

Garneau *et al.* [151] used an e-nose to investigate a problem relating to the quality of pulp and paper which is sometimes affected by the proportions of black spruce (*Picea mariana*), balsam fir (*Abies balsamea*) and jack pine (*Pinus banksiana*) present in wood chips used as raw materials. A rapid analytical method capable of differentiating wood of these three species of conifers was a prerequisite to developing a procedure to determine the proportions. This differentiation was achieved through the use of the Cyranose 320 electronic nose. Complete discrimination of the sapwood and heartwood of these three conifers was achieved.

### 6.8. Medical Pathology

Modern medicine faces the problem and challenge of achieving effective disease diagnoses through early detections of pathogenesis or disease conditions in order to facilitate the application of rapid treatments, but at the same time dramatically reducing the invasiveness of diagnostic treatments. Chemical analysis of human biological samples, such as breath, blood, urine, sweat and skin, are the most common means of diagnosing most pathological conditions. As summarized in the “metabolic profile concept” described by Jellum *et al.* [152], current clinical chemistry is largely limited to investigations of the composition of human fluids [153]. It is well known that pathogenic microbial species produce a wide range of VOCs, and the diagnostic potential of pathogen recognition through analysis of secondary microbial metabolites was recognized and considered theoretically possible as early as the 1960s [154]. However, the use of VOC chemical analyzers, such as GC or GC/MS, is still very expensive, requires highly-skilled personnel and is time consuming to the extent of precluding early diagnoses. The connection between differences in the aroma of diseased vs. healthy human tissues and diagnostic detection of human pathogenesis is supported by studies using the extraordinarily keen olfactory abilities of well trained dogs whose sense of smell is one million times greater than human’s in the ability to detect melanoma tissues [155], bladder cancer [156], as well as lung and breast cancers [157].

Many medical researchers have published experimental data in the last ten years to demonstrate the feasibility of using the electronic nose to diagnose human diseases and to identify many different pathogenic microorganisms through the detection of the VOCs they emit both in vitro and *in vivo* [158]. The earliest works were done on plate cultures of well known pathogenic microorganisms. Gibson *et al.* [159] correctly classify 12 different bacteria and human-pathogenic yeasts with a precision of 93.4%. These results opened the door to many others. Dutta *et al*. [160] used the Cyranose 320 e-nose for in situ diagnostic analysis of patients in the hospital environment to identify three different strains of *Staphylococcus aureus* bacteria responsible for ear, nose, and throat infections. They used an innovative object-oriented data clustering approach by combining PCA based on three-dimensional scatter plots using Fuzzy C Means (FCM) and a self-organizing map (SOP) network. The results showed that the Cyranose 320 e-nose was capable of identifying the three bacterial aroma-subclasses of *S. aureus* strains with up to 99.69% accuracy. These results provided support for a new rigorous tool and method for the early detection and identification of *S. aureus* infections in hospitals. This research was related to work by Gardner *et al.* [161] who used an electronic nose to predict the class and growth phase of two potentially pathogenic microorganisms: *Escherichia coli* and *S. aureus*. Head spaces were examined by using an array of six different metal oxide semi-conducting gas sensors, classified by a multi-layer perception (MLP) with a back-propagation learning algorithm. The best MLP was found to successfully classify 100% of unknown *S. aureus* samples and 92% of unknown *E. coli* samples. The growth phase of the bacteria, previously determined from optical cell counts, was predicted from head space samples with an accuracy of 81%. According to these results, the type and growth phase of pathogenic bacteria could be correctly predicted with the use of an electronic nose.

Some highly pathogenic gastroesophageal bacteria were correctly discriminated by Pavlou *et al.* [162] whose trials involved analysis of headspace volatiles from complex broth cultures of *Staphylococcus aureus*, *Klebsiella spp*., *Helicobacter pylori* (the most common ulcer-causing pathogen) and *Enterococcus faecalis*. They successfully identified these bacteria at a diluted concentration of 10^7^ CFU mL^−1^. More recently, other in vitro studies have reported the feasibility of recognizing several strains of two anaerobic bacteria (14 strains of *Clostridium* spp. and 12 strains of *Bacteroides fragilis*) and two fecal pathogens (*E. coli* and *Salmonella typhimurium*) by the use of an electronic nose [163,164]. Moens *et al.* [165] were able to dramatically reduce the time between isolation and identification of ten clinically important microorganisms (*Pseudomonas aeruginosa*, *E. coli*, *Klebsiella pneumoniae*, *Enterobacter aerogenes*, *Proteus vulgaris*, *S. aureus*, *Streptococcus pneumoniae*, *E. faecalis*, *Candida albicans*, *Aspergillus fumigatus*) using an electronic nose.

Further work on the development of microbe discrimination and classification in culture plates of pathogenic bacteria has been done by the sampling and analysis of biological fluids of diseased patient volunteers. Chandiok *et al.* [166] obtained good early results in diagnosing bacterial vaginosis from vaginal swabs of 68 women attending a genitourinary clinic. The conventional time-consuming sensing approach was based on pH change, presence of clue cells, visual analysis of vaginal discharge and human perception of amine-odor after addition of KOH. These methods were arguably unpleasant and very subjective. Hay *et al.* [167] were able to dramatically improve the accuracy of the same pathological diagnosis using a commercially available electronic nose in two separate trials. They found the e-nose increased the sensitivity and specificity of the diagnosis by 76–81% compared with Amsel criteria, and 77–83% compared with Gram-stain tests.

Urinary tract infections have been thoroughly investigated by Di Natale *et al.* [168] and Aathithan *et al.* [169]. Pavlou *et al*. [163] proposed the use of the electronic nose as a potential diagnostic tool for patients affected with kidney diseases, by distinguishing traces of blood in urine samples, and for the rapid identification of *E. coli*, *Proteus* spp. and *Staphylococcus* spp. infections at very high levels of confidence. Aathithan *et al.* [169] analyzed 534 clinical urine specimens of which 21 % had significant bacteriuria indications. The sensitivity and specificity of the electronic nose compared with conventional cultural counts were 83.5% and 87.5% respectively, but the e-nose diagnoses were done at significantly lower costs. Boilot *et al.* [170] classified six bacteria responsible for eye infections and ENT (ear, nose and throat) disease with an accuracy of 97.3% and 97.6% respectively using a commercially-available electronic nose. They were able to discriminate between pure laboratory cultures containing a fixed volume of bacteria in suspension and blood agar plates used to culture samples collected from diseased patients.

Lykos *et al.* [171] proposed sensorial analysis as an alternative method to identify bacteria from blood cultures of patients with bacteremia and septicemia (caused by *E. coli*, *Pseudomonas aeruginosa*, *S. aureus* and *E. faecalis*) instead of the conventional, subculturing procedures done on diagnostic plate media. Blood analysis via the electronic nose was used by Fend *et al.* [172] to monitor and quantify dialysis dosage in patients undergoing regular renal dialysis following kidney failure. The dialysis dose is a function of the urea reduction rate and was detectable by the electronic nose that was capable of discriminating pre-dialysis from post-dialysis blood. This tool could be used for on-line dialysis monitoring.

Conceptually, the electronic nose has interesting applications in the sensorial analysis of human breath to potentially provide quick diagnosis of many diseases. In the case of pneumonia diagnosis, Hockstein *et al.* [173] discriminated between diseased and non-diseased patients with an accuracy as high as 91.6%. The severity of asthma also was investigated by use of the electronic nose in young and older patients with mild and severe asthma [174]. Smell prints of patients with moderate asthma symptoms were fully separated from controls, whereas patients with mild and severe asthma were not as easily discriminated.

The presence of *Mycobacterium* tuberculosis, etiologic agent of tuberculosis and a world-wide major public heath problem particularly in developing countries, was investigated by Pavlou *et al.* [175] and Fend *et al.* [176], both in vitro and in situ, by means of e-nose analysis of sputum samples of diseased patients. They were able to identify the pathogen at very high levels of confidence (100% and 89%) in these respective sputum samples.

One of the most disputed yet promising application of electronic nose technologies is for the early detection and diagnosis of oncologic diseases, in particular lungs cancer. Since 1971, it has become well known that hundreds of VOCs are present in the human breath [177], and that some of these compounds are associated with certain diseases [178–180]. O’Neil *et al.* [181] reported higher levels of alkanes (hexane and methylpentane in particular) in the breath of patients affected by lung cancer; later confirmed by Philips *et al.* [182,183]. The initial work to evaluate the feasibility of the electronic nose in detecting these cancer-marker compounds was done by Di Natale *et al.* [184]. They collected breath samples from 60 individuals: 35 of which were affected by lung cancer, 9 had just had surgical therapy and 18 were used as controls. Two more individuals were measured before and after surgical therapy. The electronic nose could successfully detect 100% of lung cancer affected patients, 94% of controls and 44% of post-surgery patients (the others were classified as healthy controls). In this study, Di Natale used a quartz microbalance gas sensors electronic nose as did Yu *et al.* [185] and Chen *et al.* [186] who confirmed that the electronic nose could successfully identify eleven diagnostic VOCs that were validated as indicators or chemical markers of lung cancer. They found this e-nose performed at 71.4% sensitivity and 91.4% specificity for detecting lung cancer, with positive and negative predictive values of 66.6% and 94.5%, respectively.

A recent paper by Gendron *et al.* [187] reported on the in vitro discrimination of tumor cell lines by use of an electronic nose. Cells from both tumor (adenocarcinoma, squamous cell carcinoma, mesothelioma) and normal healthy cell lines were suspended in saline solution and analyzed by the Cyranose 320 e-nose (Smiths Detection, Pasadena, CA). This electronic nose could distinguish between cancer cell lines derived from skin lesions and was later demonstrated to have good sensitivity towards VOCs emitted from skin portions of patients affected by melanomas [153]. Some other demonstrated applications of the electronic nose in the field of clinical medicine include the possibility of monitoring microbial metabolites released from superficial wounds and burns in order to detect the presence of bacterial growth [188], upper-respiratory infections in the field of rhinology [189], the diagnosis of diabetes [190], and to distinguish cerebrospinal fluid from serum [191,192]. Many other developing medical applications for the electronic nose recently have shown promise including the diagnosis of ventilator-associated pneumonia [173,193–194], detection of cerebrospinal fluid [191,192], identification of bacterial pathogens [195,196], early screening for the presence of many different types of cancers [183,197], and breath analyses for detection of various diseases [198], toxin exposure [199], and radon ingestion [200].

The research and development (R&D) of electronic-nose applications in the biomedical field is growing at such a phenomenal rate that the development of e-nose applications in other fields, in some cases, may be suffering by comparison as a result of the increasing demand for problem solutions to the many and varied medical needs of modern societies. The stronger emphasis of research priorities and funding for the development of new e-nose technologies in the medical industry is related to the higher cost of detection instruments needed for disease diagnoses, the increasing demand for such instruments at large numbers of medical hospitals/clinics and research facilities, the greater availability of funding for instrument purchases, the higher visibility of biomedical needs and new diagnostic discoveries, and the concomitant shift in emphasis of R&D activities of commercial companies that develop electronic noses in response to these social, economic, and profit-motivated pressures. The result is that some companies that have formerly developed e-nose technologies for diverse applications in many industries have shifted their entire R&D programs toward biomedical applications. Electronic nose instruments developed for diagnostic medical applications are considerably higher priced and more lucrative for commercial development. Thus, there are many motivations for e-nose producers to specialize in the field of medical diagnostics.

### 6.9. Chemistry and Chemical Detection

The capabilities of utilizing certain EAD technologies for the detection, identification, classification and characterization of individual compounds or specific classes of chemicals present in simple or complex vapor mixtures has been realized for numerous applications in the field of chemistry, including chemical analysis, sensor-design research and for the development of new chemical-detection tools useful for solving many practical problems requiring highly-specialized chemical detection methods. Consequently, most chemical detection methods and tools utilizing chemical sensor arrays tend to be developed for very specific applications due to the specialized nature of individual detection problems. Albert *et al.* [201] provided a thorough review of cross-reactive type chemical sensor array technologies, developed prior to 2000, that utilize pattern recognition methods as opposed to specific-analyte sensors for analyte recognition and identification. Considerable research completed since 2000 has provided more details of advances in chemical sensor arrays including sensor capabilities, designs, responses and performance under various conditions and in specific areas of application. Much research in this area has been done by Dr. Nathan S. Lewis and numerous colleagues utilizing carbon-black polymer composite (CBPC) vapor detectors as outlined in the following discussions.

Matzger *et al.* [202] examined two combinatorial strategies to obtain sensor diversity from a limited number of chemical feedstocks used in constructing conducting polymer composite arrays. One approach involved the use of a series of block copolymers synthesized from combinations of monomer feedstocks, and the other method utilized a series of plasticizers to modify the properties of a base polymer vapor detector. They found both methods yielded unique sorption data with differing array response patterns for various analytes, suggesting that chemically diverse detector arrays with variable vapor-response properties could be formed from a limited number of feedstock solutions.

Briglin *et al.* [203] investigated spatiotemporal response properties and geometric factors affecting optimization of signal/noise (S/N) performance of a CBPC detector array. Response behaviors of detectors having a variety of different geometric form factors indicated that there is an optimum detector film volume that will produce the highest S/N ratio for a given carbon black polymer composite when exposed to a fixed volume of sampled analyte. Different form factors of a given detector film along with specific analyte flow paths provided very different detection performances for different analyte vapors. Useful information on the composition of analytes and analyte mixtures was obtained from changes in detector signals in response to variations in analyte flow rates. They also reported that S/N data allow comparisons between the detection limits of several polymer/analyte combinations using two different modes of signal transduction: frequency shifts in SAW devices and electrical resistance changes in composites of carbon black and insulating-organic conducting polymer devices.

Pardo *et al.* [204] compared two classification algorithms, Fisher’s linear discriminant (FLD) and a multilayer perception neural network (MLP), to directly compare the methods relative to respective capabilities of differentiating response patterns from CBPC arrays. Comparisons were done using five types of tasks that cause analyte classification problems of varying difficulty. Both methods yielded comparable performances on straightforward analyte classification tasks, whereas the MLP technique yielded better performance on tasks that involved non-linear classification boundaries. Optimal test-set performance distribution was found to be significantly better with MLP than with FLD (85% vs. 57% correct classification rate).

Several studies have examined and compared the detector responses of conducting polymer CBPC-based electronic noses to mammalian olfactory systems. Lewis [205] indicated that the mammalian olfactory system uses an array of broadly cross-reactive nerve receptors in which each analyte elicits a response from an array of receptors and each receptor responds to a collection of odorants. He found the mammalian system to be strongly analogous to the broadly cross-reactive vapor sensors of conducting polymer e-noses that robustly classify, identify, and quantify diverse organic vapor analytes based on the collective responses of the sensor array, even though no individual sensor responds selectively to a particular analyte. Burl *et al.* [206] tested the ability of a conducting polymer CBPC e-nose to accurately predict the perceived quality of an odorant as reported by human panelists using “human perceptual space” or English language descriptors used to describe odors. Various data analysis techniques were used to determine mappings from e-nose measurements to these odor descriptors. Some mapping models yielded cross-validated predictions that correlated well with the human data, but none of the models accurately predicted the human values for more than a few descriptors. Doleman and Lewis [207] compared the odor detection thresholds and odor discriminabilities of a CBPC electronic nose to the olfactory characteristics of monkeys and humans. Comparisons were only made for volatile organic vapors as opposed to aroma active odorant vapors. The trends in odor detection thresholds of the e-nose were very similar to those exhibited by humans. Discrimination performance of the e-nose and mammals increased as the compounds of the odorant pairs became more structurally dissimilar. However, the electronic nose exhibited significantly better discriminability than humans or monkeys for the odorant pairs evaluated.

Recent efforts to improve e-nose sensor design have involved the development of chemiresistors, modification of sensor film thickness and composition, improvements in sensor response time, adjustments of array size (numbers of chemically different detectors in the array), and refinements in analyte classification performance. Thin-film chemiresistive vapor sensors, formed from composites of carbon black and low volatility nonpolymeric organic molecules (propyl gallate, lauric acid, and dioctyl phthalate, metallophthalocyanines, etc.), have the advantages of operation at relatively low power consumption levels (0.1–1 mW), comparatively simple compact design aptly suitable for miniaturization and portability, compatibility with very large-scale integration (VLSI) processing, rapid response time, rapid reversible changes in electrical resistance response of the sensing films, and the capability of detecting inorganic gases as well as organic vapors [208,209]. Furthermore, such sensors can be deposited onto a variety of electrodes and insulating materials, and can be fabricated in a wide variety of form factors to optimize S/N ratios to produce desired sensor array configurations [210]. Using nonpolymeric sorption phases allow fabrication of sensors having a high density of randomly-oriented functional groups providing excellent discrimination between analytes [209]. Increasing the density of the functional groups in the sorption material increases the amount of vapor sorption resulting in increased detector sensitivity. Briglin and Lewis [211] discovered the response times of polyethylene-*co*-vinyl acetate (PEVA)-carbon black sensors were proportional to the square of the film thickness. Temporal modulation of the analyte concentration permitted ready separation of the signal into its analyte-induced component, leading to a reduction in the noise error. Burl *et al.* [212] found the vapor classification performance of CBPC arrays was a function of the number and type of detectors in the array. Classification performance increased or did not significantly decrease as the number of chemically-different detectors in the array increased.

There has been a growing interest in the development of vapor detectors sensitive to carboxylic acids, particularly volatile fatty acid by-products derived from the metabolic pathways of certain pathogenic bacteria, because these compounds are frequently released into the lungs and expelled by humans having certain diseases caused by these microbes. Thus, the exhalation of specific fatty acid mixtures is indicative and diagnostic of specific bacterial species, providing a means of identifying and classifying disease-causing agents (without bacterial culturing) in order to prescribe appropriate treatments. CBPC vapor detectors containing linear polyethylenimine (*l*-PEI) as the insulating component with amino-terminated dendrimer-carbon black composites exhibited an enhancement in detection sensitivity of ∼10^3^ for volatile carboxylic acids [213]. Protonated carboxylato-terminated and amino-terminated composites showed a ∼10^3^–10^4^ increase in sensitivity for detection of volatile amines. Sensitive detection and robust discrimination between various volatile organic acids was achieved with relatively low sensor responses to nonacidic organic vapors or water vapor [214]. The mechanism of enhanced sensor sensitivities towards volatile carboxylic acid vapors were quantified based on relative contributions of electrical percolation effects, increases in analyte sorption, and charge-induced swelling effects of the sorptive polymer films [215].

New information from spatiotemporal-response data derived from cross-reactive sorption-based sensor arrays indicates that cross-reactive vapor sensors are not only capable of correctly identifying and quantifying vapor mixture components, but also provide information on physicochemical properties of analytes, such as degree of unsaturation of carbon chains, dipole moment, molecular weight, number of hydrogen atoms and type of aromatic rings present [216]. This information is relevant for applications of a semi-selective array of vapor sensors in situations where no prior knowledge of analyte identity is available and when there is no assurance that the test analyte is contained within the reference library of known analyte responses previously compiled from the detector array. Linear sensor arrays, composed of small-molecule carbon black composite chemiresistors placed in a low-headspace volume chamber with vapor mixtures delivered at low flow rates, enabled the correct identification and quantification of vapor mixtures containing up to five components using only four chemically-different sorbent films [217].

Optical fiber-based sensor arrays of various types recently have been developed with a wide diversity of chemical applications owing to the extreme versatility of sensor designs and configurations that are possible, the miniature size that facilitates faster response, and the ability to simultaneously acquire multiple optical properties from chemical analytes. Fiber-optic sensors have been used for the development of nucleic acid probes for various genomic applications, microbial pathogen detection methods, and live cell-based sensors for monitoring specific chemicals and toxins in the environment [218]. Kuang *et al.* [219] developed an *Escherichia coli* living bacterial cell-based optical fluorescence biosensor array to detect the presence of genotoxins (toxins that cause DNA damage) in the environment. Each optical fiber in the microwell array has its own light pathway, enabling thousands of individual bacterial cell responses to be monitored simultaneously. The *E. coli* cells are arranged singly in each microwell at the end of an imaging fiber bundle. The live cells carry a recA::gfp fusion plasmid that undergoes gene transcription upon DNA damage to produce the RecA protein that mediates the self-cleavage of LexA protein, leading to the induction of the SOS regulon. The GFPmut2 [220] protein variant, cloned from the jellyfish *Aequorea victoria* [221], was used as the reporter protein because it is intrinsically fluorescent. The imaging microwell array is mounted on an epifluorescence microscope focused on the proximal end of the fiber bundle. A charge-coupled device (CCD) camera acquires fluorescence intensity emissions (520 nm wavelength) from individual cells through an objective lens which are recorded immediately after exposing the cell array to a test medium, and then analyzed by specialized software. A similar optical imaging fiber bundle microwell array utilizing a live-cell system was developed by Kuang and Walt [222] for testing new drug candidates on multiple cell lines in order to understand how cells respond dynamically to drugs and how cellular pathways respond cooperatively to drugs in individual cells. This information is needed in the development of new drug regimens with multiple biologically-active agents designed to act synergistically on multiple biochemical targets.

Fiber-optic microarray systems also have been developed as DNA oligonucleotide probes to detect specific harmful microbes in food or in environmental samples. Ahn and Walt [223] produced a fiber optic DNA microarray, prepared by randomly distributing DNA probe-functionalized microspheres (3.1 μm diameter) into microwells created by etching optical fiber bundles, for the detection of *Salmonella* species in foods. Hybridization of the probe microspheres to *Salmonella* spp.-target DNA was performed and visualized using Cy3-labeled secondary probes in a sandwich-type assay format. Song *et al.* [224] developed a similar system composed of 18 species-specific probe microsensors to identify biological warfare agents. By employing multiple 50-mer DNA probes and many replicates for each biological warfare agent, the potential for false-negatives and false-positive results were reduced and detection confidence increased with lower S/N ratios.

Recent mini-reviews of fiber optic microarray technologies summarize a wide range of sensor types, designs, target analytes, and potential applications in diverse fields and industries. Monk and Walt [225] discussed optical fiber-based biosensors that are classified according to the nature of the biological recognition element used for sensing: including enzymes, antibody/antigen (immunoassay), nucleic acid, whole cell, and biomimetic types that are use for analytes ranging from metals and chemicals to physiological materials. Epstein and Walt [226] describe applications in combined-image sensing on live cells, corrosion monitoring, high-density sensing, artificial olfaction, biomonitoring, oligonucleotide detection, molecular beacons, aptamers, and cell-based systems. Szunerits and Walt [227] review the use of optical fiber bundles combined with electrochemistry for chemical imaging. Fluorescent imaging of biological processes is widely used because of the variety of fluorescent dyes, enzymes and other labeling biomolecules that are readily available commercially for applications in biochemistry, biophysics, analytical chemistry, and clinical diagnostics. Walt [228] summarized the use of miniature biosensors and sensor arrays as medical diagnostic devices that may be used closer to the patient similar to other point-of-care devices. These tools will eventually replace most conventional and expensive analytical equipment used in disease diagnoses because they will be able to detect clinically-important analytes more quickly and with greater specificity and certainty.

Fluorescent microbead high-density multisensor arrays also may be used in artificial olfaction for odor discrimination and classification of chemical analytes. Albert and Walt [229] examined two sensor-response approaches for odor discrimination. In the first approach, sensor responses from individual microsensors were separated (decoded array) and independently processed. In the second approach, sensor-response profiles from all microsensors within the entire array (sensor ensemble) were combined to create one response per odor stimulus (nondecoded array). Both signal-extraction approaches showed comparable odor discrimination rates, but the ensemble approach streamlined system resources without decreasing system performance. Bencic-Nagale and Walt [230] discovered two ways of extending the longevity of fluorescence-based multisensor arrays that are degraded by photobleaching. Photobleaching was overcome by limiting the excitation light power and gradually increasing the power at a rate comparable to the rates of sensor photobleaching, and by illuminating subsections of the array through an optical slit. These two improvements resulting in a 90-fold increase in the time during which the sensor array responded reproducibly to a diverse group of vapors, and worked much better than antifading agents, use of fluorescent polymers with increased photostability, or ratiometric measurements.

## 7. Conclusions

A universal electronic nose capable of identifying or discriminating any gas sample type with high efficiency and for all possible applications has not as yet been built. This fact is largely due to the selectivity and sensitivity limitations of e-nose sensor arrays for specific analyte gases. Electronic noses are not designed to be universally appropriate sensor systems for every conceivable gas-sensing application nor are they capable of serving every possible analytical need. Thus, the suitability of an electronic nose for a specific application is highly dependent on the required operating conditions of the sensors in the array and the composition of the analyte gases being detected. A proper selection of an appropriate e-nose system for a particular application must involve an evaluation of systems on a case by case basis. Some key considerations involved in e-nose selection for a particular application must necessarily include assessments of the selectivity and sensitivity range of individual sensor arrays for particular target analyte gases (likely present in samples to be analyzed), the number of unnecessary redundancy sensors with similar sensitivities, and various operational requirements such as run speed or cycle time, recovery time between samples, data analysis and result-interpretation requirements. These operational considerations for e-nose selection are in addition to normal practical considerations such as instrument price, operation and maintenance costs, portability requirements, and necessary ease-of-use by the intended operators. Most e-noses are not fully automated in their operation, but require some data processing and statistical analyses to obtain useable results. Consequently, the process of electronic-nose sensing of analyte gases is a bit of an art form involving not only proper instrument and sensor-array selection, but also some experience and training in proper e-nose operational protocols; although training requirements for electronic noses are much less rigorous than those for complex analytical instruments. Of course, combined-technology electronic noses require more training and skill for operation than traditional single-technology instruments.

Artificial or electronic noses with diverse sensor arrays that are differentially responsive to a wide variety of possible analytes have a number of advantages over traditional analytical instruments. Electronic nose sensors do not require chemical reagents, have good sensitivity and specificity, provide rapid results, and allow non-destructive sampling of odorants or analytes [231]. Furthermore, e-noses generally are far less expensive than analytical systems, easier and cheaper to operate, and have greater potential for portability and field use compared with complex analytical laboratory instruments. Thus, electronic noses have far greater potential to be used eventually by unskilled consumers for innumerable practical applications in residential and public settings. Some disadvantages of e-nose sensing include problems with reproducibility, recovery, negative effects of humidity and temperature on the sensor responses, and inability to identify individual chemical species with sample gases. Thus, electronic noses will never completely replace complex analytical equipment or odor panels for all applications, but offer quick real-time detection and discrimination solutions for applications requiring accurate, rapid and repeated determinations. Such applications are becoming increasingly common and required for highly-mechanized industrial manufacturing processes. Furthermore, the real time rapid-analysis capabilities of new portable e-noses, currently under development by various manufacturers, are not only required but expected operating capabilities needed to accommodate the many fast-paced activities and mechanized processes of modern society.

The aforementioned summaries of commercial applications, developed for electronic-nose devises within the past twenty years, have only covered some of the more interesting, compelling and perhaps most beneficial uses of e-noses under current operation today. The intent of this paper was by no means aimed at providing a comprehensive review of all known e-nose applications that have been developed. Such an effort would require a much more extensive treatise far beyond the scope of this current summary. Obviously, many other applications of electronic noses exist that were omitted from being mentioned here. Nevertheless, a brief mention of on-going and future developments of electronic-nose technologies is warranted here in order to provide a greater appreciation of the breadth of research projects and operational programs that are involving routine uses and further improvements in e-nose applications.

New emerging technologies are continually providing means of improving e-noses and EAD capabilities through interfaces and combinations with classical analytical systems for rapid discrimination of individual chemical species within aroma mixtures. E-nose instruments are being developed that combine EAD sensors in tandem with analytical detectors such as with fast gas chromatography (FGC) [20]. More complicated technologies such as optical gas sensor systems also may improve on traditional e-nose sensor arrays by providing analytical data of mixture constituents [232]. These technologies will have the capability of producing recognizable high resolution visual images of specific vapor mixtures containing many different chemical species, but also quantifying concentrations and identifying all compounds present in the gas mixture. Similar capabilities for identifying components of solid and liquid mixtures may be possible with devices called electronic tongues [233,234]. Several recent reviews provide summaries of electronic tongue technologies and discuss potential applications for food analyses [235–237].

The potential for future developments of innovative e-nose applications is enormous as researchers in many fields of scientific investigation and industrial development become more aware of the capabilities of the electronic nose. The current trend is toward the development of electronic noses for specific purposes or a fairly narrow range of applications. This strategy increases e-nose efficiency by minimizing the number of sensors needed for discriminations, reducing instrument costs, and allowing for greater portability through miniaturization. New potential discoveries in this relatively new sector of sensor technology will continue to expand as new products, machines, and industrial processes are developed. These discoveries will lead to the recognition of new ways to exploit the electronic nose to solve many new problems for the benefit of mankind.

## Figures and Tables

**Table 1.** Examples of primary aroma categories proposed by Amoore [42,43] in 1964.

**Primary aromas**	**Example compound**	**Chemical structure**	**Common source**
Camphoraceous	camphor	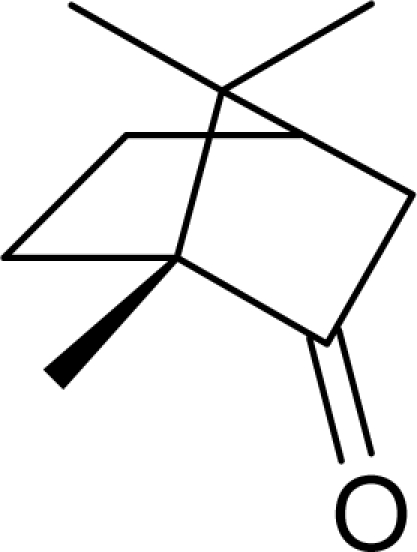	mothballs
Ethereal	ethylene dichloride	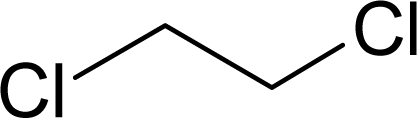	dry cleaning fluid
Floral	phenylethyl methyl ethyl carbinol	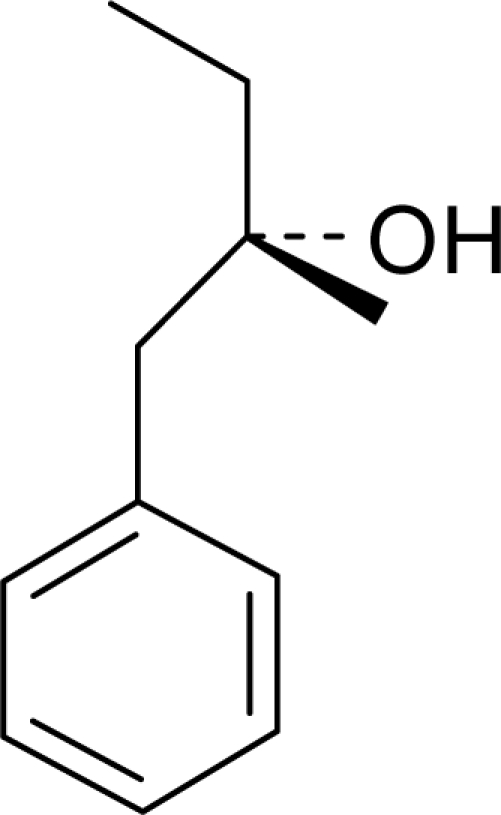	rose fragrance
Musky	ω-pentadecalactone	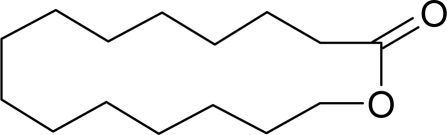	angelica root oil
Pepperminty	menthone	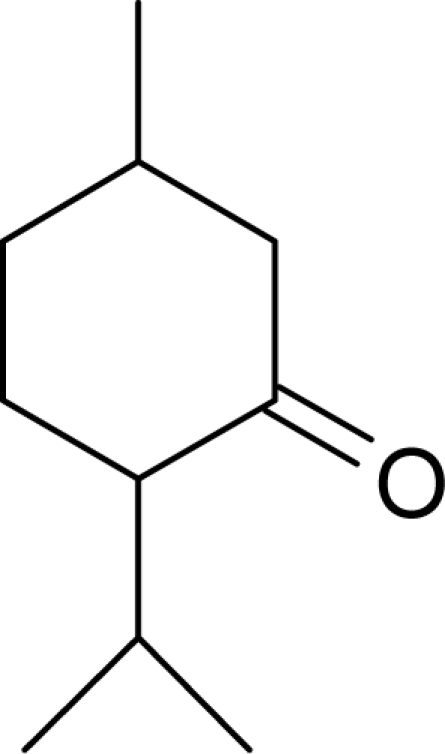	peppermint oil
Pungent	formic acid	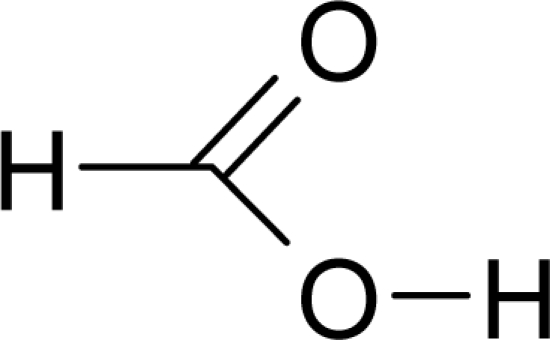	ant secretion
Putrid	butyl mercaptan	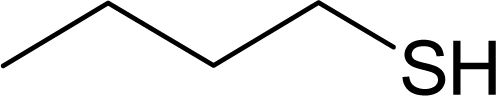	skunk odor

**Table 2.** Range of human detection thresholds for some common odorants in air.

**Odorant**	**Aroma type or source**	**Chemical structure**	**Human detection Threshold (mg dm^−3^)†**
Benzaldehyde	bitter almond	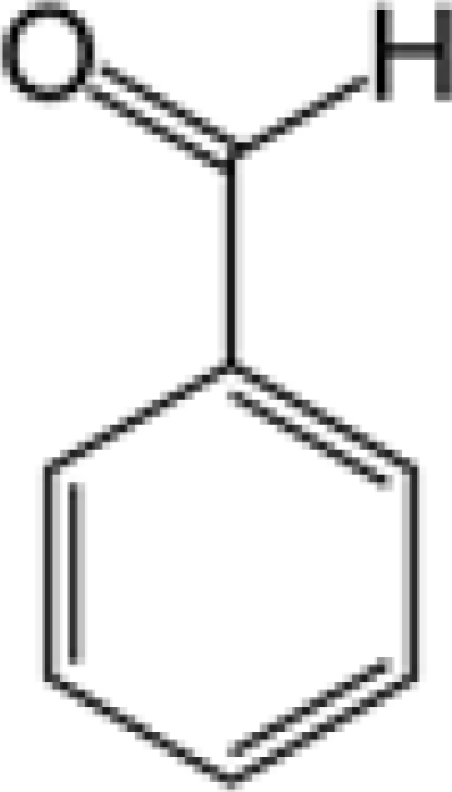	3.0 × 10^−3^
Butyric acid	rancid butter	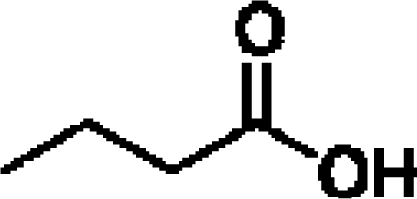	9.0 × 10^−3^
Citral	lemon	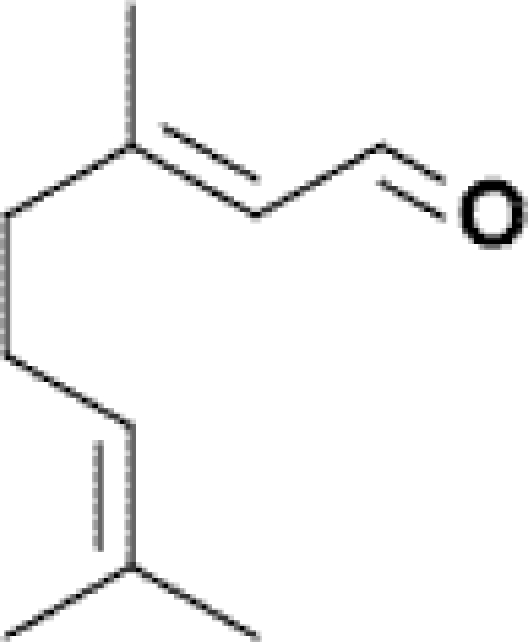	3.0 × 10^−6^
Ether	ether	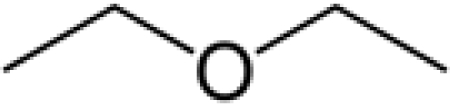	5.8
Ethyl butyrate	fruity	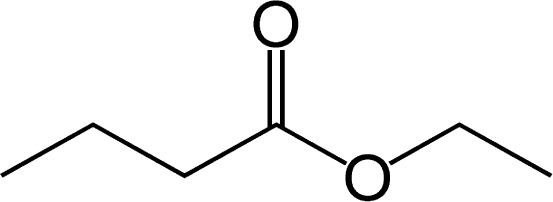	1.0
Limonene	lemon	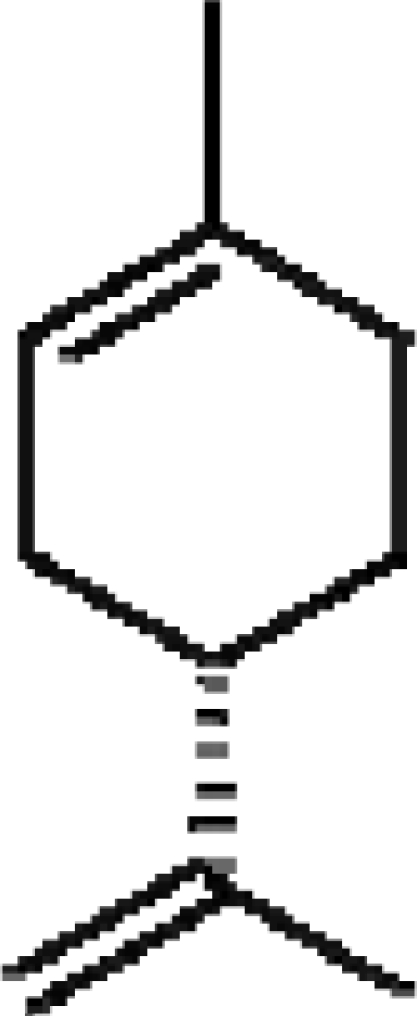	0.1
Methyl salicylate	wintergreen	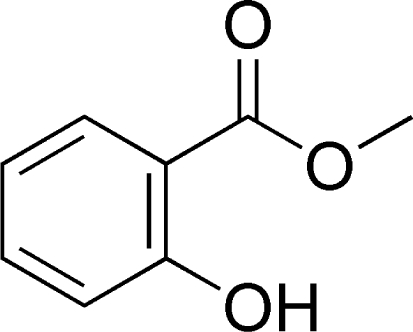	0.1
Pyridine	pungent	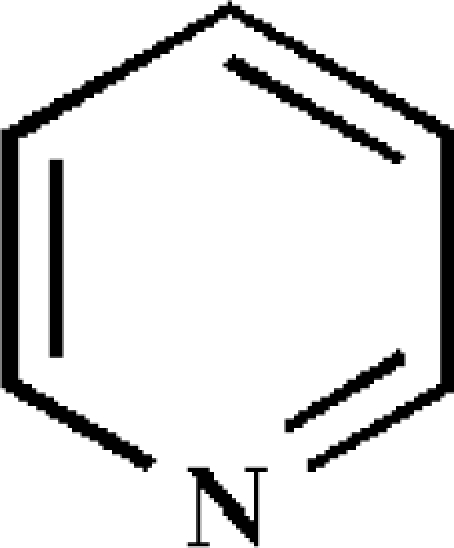	3.0 × 10^−2^

† A human detection threshold concentration of 0.1 mg dm^−3^ for a gas or particulate odorant in dry air is equivalent to 77.1 parts per million (ppm) at standard temperature and pressure (STP).

**Table 3.** Types and mechanisms of common electronic-nose gas sensors.

**Sensor type**	**Sensitive material**	**Detection principle**
Acoustic sensors: Quartz crystal microbalance (QMB); surface & bulk acoustic wave (SAW, BAW)	organic or inorganic film layers	mass change (frequency shift)
Calorimetric; catalytic bead (CB)	pellistor	temperature or heat change (from chemical reactions)
Catalytic field-effect sensors (MOSFET)	catalytic metals	electric field change
Colorimetric sensors	organic dyes	color changes, absorbance
Conducting polymer sensors	modified conducting polymers	resistance change
Electrochemical sensors	solid or liquid electrolytes	current or voltage change
Fluorescence sensors	Fluorescence-sensitive detector	fluorescent-light emissions
Infrared sensors	IR-sensitive detector	Infrared-radiation absorption
Metal oxides semi-conducting (MOS, Taguchi)	doped semi-conducting metal oxides (SnO2, GaO)	resistance change
Optical sensors	photodiode, light-sensitive	light modulation, optical changes

**Table 4.** A partial list of gases that have been detected using electrochemical (EC) sensors.

**Gas detected**	**Formula**
Acetaldehyde	CH_3_CHO
Acetylene	C_2_H_2_
Acrylic acid	C_2_H_3_COOH
Ammonia	NH_3_
Antimony pentachloride	SbCL_5_
Arsine	AsH_3_
Boron trichloride	BCL_3_
Boron trifluoride	BF_3_
Bromine	Br_2_
Butadiene	(C_2_H_3_)_2_
Butyl acrylate	C_2_H_3_COOC_4_H_9_
Carbon monoxide	CO
Chlorine	Cl_2_
Chlorine dioxide	ClO_2_
Chlorine trifluoride	ClF_3_
Diborane	B_2_H_6_
Dichlorosilane	SiH_2_Cl_2_
Diethyl aminoethanol	(C_2_H_5_)_2_NC_2_H_4_OH
Dimethyl amine	(CH_3_)_2_NH
Dimethyl sulfide	(CH_3_)_2_S
Epichlorohydrin	C_2_H_2_OCH_2_Cl
Ethanol	C_2_H_5_OH
Ethylene oxide	C_2_H_4_O
Ethylmercaptan	C_2_H_5_SH
Fluorine	F_2_
Formaldeyde	HCHO
Germanium tetrahydride	GeH_4_
Hydrogen	H_2_
Hydrogen bromine	HBr
Hydrogen chloride	HCl
Hydrogen cyanide	HCN
Hydrogen fluoride	HF
Hydrogen peroxide	H_2_O_2_
Hydrogen sulfide	H_2_S
Isopropanol	(CH_3_)_2_CHOH
Isopropyl amine	(CH_3_)_2_CHNH_2_
Isopropyl mercaptan	(CH_3_)_2_CHSH
Methanol	CH_3_OH
Methyl mercaptan	CH_3_SH
Methyl methalacrylate	CH_2_=C(CH_3_)COOCH_3_
Monomethylamine	CH_3_NH_2_
Morpholine	C_4_H_8_ONH
Nitrogen dioxide	NO_2_
Nitrogen monoxide	NO
Oxygen	O_2_
Phosgene	COCl_2_
Phosphorus trichloride	PCl_3_
Phosphorus trihydride	PH_3_
Phosphoryl chloride	POCl_3_
Propylene	CH_3_CH=CH_2_
Propylene oxide	C_3_H_6_O
n-propyl mercaptan	C_3_H_7_SH
Sulphur dioxide	SO_2_
Silicon tetrachloride	SiCl_4_
Tetrahydrothiophene	C_4_H_8_S
Thionyl chloride	SOCl_2_
Titanium tetrachloride	TiCl_4_
Trichlorosilane	SiHCl_3_
Tungsten hexafluoride	WF_6_
Tin tetrachloride	SnCl_4_

**Table 5.** Summary of advantages and disadvantages of e-nose sensor types.

**Sensor type**	**Advantages**	**Disadvantages**
Calorimetric or catalytic bead (CB)	Fast response and recovery time, high specificity for oxidized compounds	High temperature operation, only sensitive to oxygen-containing compounds
Catalytic field-effect sensors (MOSFET)	Small sensor size, inexpensive operating costs	Requires environmental control, baseline drift, low sensitivity to ammonia and carbon dioxide
Conducting polymer sensors	Ambient temperature operation, sensitive to many VOCs, short response time, diverse sensor coatings, inexpensive, resistance to sensor poisoning	Sensitive to humidity and temperature, sensors can be overloaded by certain analytes, sensor life is limited
Electrochemical sensors (EC)	Ambient temperature operation, low power consumption, very sensitive to diverse VOCs	Bulky size, limited sensitivity to simple or low mol. wt. gases
Metal oxides semi-conducting (MOS)	Very high sensitivity, limited sensing range, rapid response and recovery times for low mol. wt. compounds (not high)	High temperature operation, high power consumption, sulfur & weak acid poisoning, limited sensor coatings, sensitive to humidity, poor precision
Optical sensors	Very high sensitivity, capable of identifications of individual compounds in mixtures, multi-parameter detection capabilities	Complex sensor-array systems, more expensive to operate, low portability due to delicate optics and electrical components
Quartz crystal microbalance (QMB)	Good precision, diverse range of sensor coatings, high sensitivity	Complex circuitry, poor signal-to-noise ratio, sensitive to humidity and temperature
Surface acoustic wave (SAW)	High sensitivity, good response time, diverse sensor coatings, small, inexpensive, sensitive to virtually all gases	Complex circuitry, temperature sensitive, specificity to analyte groups affected by polymeric- film sensor coating

**Table 6.** Some commercially available electronic noses, models and technologies.

**Instrument type**	**Manufacturer**	**Models produced**	**Technology basis**
**Single-technology (e-nose sensors only*)***	Airsense Analytics	i-Pen, PEN2, PEN3	MOS sensors
	Alpha MOS	FOX 2000, 3000, 4000	MOS sensors
	Applied Sensor	Air quality module	MOS sensors
	Chemsensing	ChemSensing Sensor array	Colorimetric optical
	CogniScent Inc.	ScenTrak	Dye polymer sensors
	CSIRO	Cybernose	Receptor-based array
	Dr. Födisch AG	OMD 98, 1.10	MOS sensors
	Forschungszentrum Karlsruhe	SAGAS	SAW sensors
	Gerstel GmbH Co.	QSC	MOS sensors
	GSG Mess- und Analysengeräte	MOSES II	Modular gas sensors
	Illumina Inc.	oNose	Fluorescence optical
	Microsensor Systems Inc	Hazmatcad, Fuel Sniffer, SAW MiniCAD mk II	SAW sensors
	Osmetech Plc	Aromascan A32S	Conducting polymers
	Sacmi	EOS 835, Ambiente	Gas sensor array
	Scensive Technol.	Bloodhound ST214	Conducting polymers
	Smiths Group plc	Cyranose 320	Carbon black-polymers
	Sysca AG	Artinose	MOS sensors
	Technobiochip	LibraNose 2.1	QMB sensors
**Combined-technology (e-nose + other types)**	Airsense Analytics	GDA 2	MOS, EC, IMS, PID
	Alpha MOS	RQ Box, Prometheus	MOS, EC, PID, MS
	Electronic Sensor Technology	ZNose 4200, 4300, 7100	SAW, GC
	Microsensor Syst.	Hazmatcad Plus	SAW, EC
		CW Sentry 3G	SAW, EC
	Rae Systems	Area RAE monitor	CB, O_2_, EC, PID
		IAQRAE	Thermistor, EC, PID, CO_2_, humidity
	RST Rostock	FF2, GFD1	MOS, QMB, SAW

**Table 7.** Examples of some industry-based applications for electronic noses.

**Industry sector**	**Application area**	**Specific use types and examples**
Agriculture	crop protection	homeland security, safe food supply
	harvest timing & storage	crop ripeness, preservation treatments
	meat, seafood, & fish products	freshness, contamination, spoilage
	plant production	cultivar selection, variety characteristics
	pre- & post-harvest diseases	plant disease diagnoses, pest identification
		detect non-indigenous pests of food crops
Airline transportation	public safety & welfare	explosive & flammable materials detection
	passenger & personnel security	
Cosmetics	personal application products	perfume & cologne development
	fragrance additives	product enhancement, consumer appeal
Environmental	air & water quality monitoring	pollution detection, effluents, toxic spills
	indoor air quality control	malodor emissions, toxic/hazardous gases
	pollution abatement regulations	control of point-source pollution releases
Food & beverage	consumer fraud prevention	ingredient confirmation, content standards
	quality control assessments	brand recognition, product consistency
	ripeness, food contamination	marketable condition, spoilage, shelf life
	taste, smell characteristics	off-flavors, product variety assessments
Manufacturing	processing controls	product characteristics & consistency
	product uniformity	aroma and flavor characteristics
	safety, security, work conditions	fire alarms, toxic gas leak detection
Medical & clinical	pathogen identification	patient treatment selection, prognoses
	pathogen or disease detection	disease diagnoses, metabolic disorders
	physiological conditions	nutritional status, organ failures
Military	personnel & population security	biological & chemical weapons
	civilian & military safety	explosive materials detection
Pharmaceutical	contamination, product purity	quality control of drug purity
	variations in product mixtures	formulation consistency & uniformity
Regulatory	consumer protection	product safety, hazardous characteristics
	environmental protection	air, water, and soil contamination tests
Scientific research	botany, ecological studies	chemotaxonomy, ecosystem functions
	engineering, material properties	machine design, chemical processes
	microbiology, pathology	microbe and metabolite identifications
